# How to Increase the Attractiveness of the Public Health Service in Germany as a Prospective Employer? Part II of the OeGD-Studisurvey

**DOI:** 10.3390/ijerph191811733

**Published:** 2022-09-17

**Authors:** Laura Arnold, Lisa Kellermann, Florian Fischer, Franziska Hommes, Laura Jung, Amir Mohsenpour, Jan M. Stratil

**Affiliations:** 1 Academy of Public Health Services, Kanzlerstraße 4, 40472 Duesseldorf, Germany; 2 Department of International Health, Care and Public Health Research Institute—CAPHRI, Faculty of Health, Medicine and Life Sciences, Maastricht University, 6211 Maastricht, The Netherlands; 3 German Network of Young Professionals in Public Health, 80539 Munich, Germany; 4 Institute of Public Health, Charité—Universitätsmedizin Berlin, 10117 Berlin, Germany; 5 Bavarian Research Center for Digital Health and Social Care, Kempten University of Applied Sciences, 87437 Kempten, Germany; 6 Division of Infectious Diseases and Tropical Medicine, Medical Faculty, Leipzig University, 04103 Leipzig, Germany; 7 Department of Population Medicine and Health Services Research, Bielefeld University, 33615 Bielefeld, Germany; 8 Department for Psychiatry, Psychotherapy and Psychosomatic Medicine, Vitos Kurhessen Kassel, 34131 Kassel, Germany

**Keywords:** public health workforce, public health services, health services administration, Germany, capacity building, job satisfaction, workforce research, workforce development, survey research methods, OeGD-Studisurvey

## Abstract

The Public Health Service (PHS) in Germany has had difficulties in recruiting enough qualified staff for years, but there is limited research on what factors drive decisions to (not) join the PHS workforce. We explored reasons for this perceived (lack of) attractiveness. We conducted two cross-sectional surveys among medical students (MS), public health students and students from other PHS-relevant fields (PH&ONM) in Germany before (2019/2020) and during the COVID-19 pandemic (2021). Both waves surveyed self-reported reasons for why students did (not) consider working in the PHS as attractive and how this could be improved, using open-question items. Qualitative and quantitative content analyses were conducted according to Mayring. In total, 948 MS and 445 PH&ONM provided valid written responses. Reasons for considering the PHS as attractive were, among others, the perception of a good work-life balance, high impact, population health focus, and generally interesting occupations. Suggestions to increase attractiveness included reducing bureaucracy, modernization/digitalization, and more acknowledgement of non-medical professionals. Among MS, reasons against were too little clinical/patient-related activities, low salary, and occupations regarded as boring. Our findings indicate areas for improvement for image, working conditions in, and institutional structures of the PHS in Germany to increase its attractiveness as an employer among young professionals.

## 1. Introduction

In recent years, the Public Health Service (PHS, in German: Oeffentlicher Gesundheitsdienst) in Germany has not been able to attract qualified young professionals in sufficient numbers [1,2]. Already in 2015, the health ministers of the German federal states had warned in a joint statement that in face of the increasing—in some cases dramatic—staff shortages and at the same time constantly growing responsibilities, urgent measures were needed to secure function in the PHS [1]. In this context, small rural health authorities in particular report difficulties in finding well-qualified professionals, including medical doctors with a specialization in public health (in German: Facharzt Oeffentliches Gesundheitswesen).

The PHS workforce in Germany is composed of multiple different professions. However, the size and composition of the PHS workforce is not assessed or recorded on a regular basis. According to a survey conducted in 2015, administrative staff accounted for 20% of the full-time equivalents, physicians accounted for 19%, followed by social pedagogues (18%), hygiene inspectors (11%), and medical assistants (10%) [3]. Health and social science professionals accounted both for less than 1% [3]. At the end of 2021, a survey was conducted, where the PHS staff consisted of 20% physicians, 52% other specialist staff and 28% administrative personnel [4].

However, detailed information on the workforce in the PHS is only publicly available for physicians through the German Medical Association (in German: Bundesärztekammer). According to their data, the number of public health specialists has decreased by 27% over the 20-year period from 1998 to 2018 (from 1072 to 784), while the overall number of individuals with any medical specialization has increased by 52% [2]. This trend is likely to become more pressing over the forthcoming years due to the demographics in the current public health workforce of the PHS [1,3,5]. The German Medical Association estimates that about three out of four physicians employed in the PHS will retire in the next 10 to 15 years [6]. Unfortunately, comparable data are not available for non-physician members of the PHW working in the PHS.

Although these challenges were long known and widely problematized [1,7,8], they only became apparent to the broader public when the PHS reached its capacity limits during the COVID-19 pandemic and received much attention as a result [9]. While the number of filled permanent job positions has increased during the course of the pandemic, a total of 8% of permanent job positions in the PHS remained unfilled at the end of 2021 [4].

Thus, given the existing staff shortage and its likely aggravation in the future, the need to increase the perceived attractiveness of the PHS as an employer is an important and urgent challenge [2,5]. In a survey of more than 13,000 medical students published in 2018, only 3.3% of the participants stated that they definitely, and 19.7% potentially, could imagine working in the PHS [10]. In this survey, only working in the pharmacological industry and for health insurance companies was a less attractive career path [10].

To overcome these issues, multiple experts and stakeholder groups pointed out perceived deficits and highlighted potential solutions, such as overcoming the salary gap, which exists between individuals with a medical degree who work in the PHS in comparison to working in health care [1,5,8,11,12,13], improving the image of the PHS in the medical community and the society at large [1,11,12], and highlighting the diverse and broad scope of activities within the PHS [11,12,13]. Furthermore, it was repeatedly proposed to improve contact with and insights into the PHS through providing internships or more exposure to the practice of the PHS during the study [7,8,11,12,13,14]. Other options for overcoming the shortage of (young) professionals include a stronger anchoring of public health-related topics in the curriculum of medical studies and other studies with potential relevance for the PHS (e.g., in the social or political sciences) [7,13,15,16], and a general improvement of research opportunities and/or strengthening public health research within the PHS [7,13].

Similar to other European countries and beyond, medical doctors take a prominent role within the current structure of the German PHS [17,18]. In this context, experts and stakeholders stressed repeatedly the need to reform the PHS in order to be more accessible to young professionals without a medical degree [7,13,19]. However, this call is in direct contrast to the statements of, for example other experts, professional medical associations and political decision makers who primarily or exclusively focus on attracting medical professionals [1,8,20,21]. To increase the attractiveness overall, experts and stakeholders—including the German National Academy of Sciences in their analysis of public health in Germany—suggested that multi- and interdisciplinary approaches that recognize and integrate a variety of relevant professions should be strengthened [7]. This would enable the PHS to fulfill its original purpose of ensuring and improving population health, and focus PHS on the underlying social determinants of health and assure health in all policies [7,13,19].

Despite the described importance, no empirical research for the German context is available thus far, neither which assesses among students and young professionals the reasons for their lack of interest in working in the PHS nor the reasons of those inclined to follow such a career path. While some empirical studies from other countries exist [22,23,24,25,26,27,28], their findings are often not directly transferable to the German context. To overcome this knowledge gap, the OeGD-Studisurvey was initiated, a comprehensive research project to analyze the interests and perceived attractiveness of the PHS in Germany as a potential employer for students and young professionals based on two national cross-sectional surveys. The logic model outlining the theoretical foundation of the project is provided in detail in the accompanying publication [29], which also describes the quantitative analysis of the OeGD-Studisurvey and discusses key findings in the context of existing training programs in Germany.

In this publication, part II of the OeGD-Studisurvey, we focus on the qualitative data collected in the two cross-sectional surveys to identify the reasons given by respondents for why or why they do not consider the PHS as an attractive career path. Based on these responses, we aim to develop suggestions for strengthening the attractiveness of the PHS.

## 2. Materials and Methods

The methods underlying the survey overall are described in more detail in the accompanying paper [29]. In the following, we will focus on the methods particular to the qualitative analysis and only describe the methods of the overall project in brief. We conducted two cross-sectional surveys to assess the expectations of students in PHS-relevant fields of study regarding their prospective employment, with a particular focus on the PHS. The first survey (wave 1) was conducted from early December 2019 to April 2020 and focused on expectations of medical students and students of fields with potential relevance for the PHS regarding their future jobs and employers. The second survey (wave 2) was conducted from June to September 2021 in order to capture potential changes in the perception and attractiveness of the PHS resulting from increased attention it received throughout the COVID-19 pandemic. Both surveys were independent of each other but addressed the same target group and were based on the same sampling procedure.

### 2.1. Study Population and Sampling Procedure

Students from all PHS-relevant and accredited study programs at state or state-recognized German universities on an advanced level (i.e., master’s degree) were eligible to participate. The invitation to participate in the study was sent to the secretariats of all 622 eligible study programs as well as to 36 separately identified local medical student councils. They were furthermore disseminated through various national public health networks and organizations. Finally, the invitation to participate in the study was shared via the official social media channels of the German Network of Young Professionals in Public Health (NOEG) and the German Medical Students’ Association (bvmd). An overview of all common abbreviations can be found in Appendix A. A more in depth description is provided in the companioning publication [29].

### 2.2. Survey Instrument and Data Collection

The survey questionnaires were developed by a multi-professional working group consisting of members of the NOEG, the Academy of Public Health Services (AOEGW), the Federal Association for Physicians in the Public Health Service (BVOEGD), and the bvmd in an iterative, interdisciplinary process and designed as online-based surveys. The online questionnaires were generated using SoSci Survey version 3.2.00 and were made available via www.soscisurvey.de. Both questionnaires can be downloaded at the Supplement Material Table S1 of the accompanying paper of the OeGD-Studisurvey [29].

A pretest with 15 participants was performed to test the comprehensibility of questions and the time needed to complete the questionnaire, and necessary amendments were subsequently made. The study was approved by the ethics committee of Bielefeld University, and all participants were asked to provide informed consent in electronic format before taking part in the surveys, on the first pages of the data collection instruments.

The surveys included open- and closed-ended questions. Beyond the ten items eliciting socio-demographic data from the respondents, the focus of this paper is primarily on the analysis of four open-ended questions used in wave 1 and wave 2: First, all study participants were asked to select whether they considered the PHS to be an attractive employer (i.e., both regarding the role of the PHS as employer as well as whether working in the PHS is considered attractive). Those who agreed with the statement were asked to state their reasons for why they considered the PHS as attractive in a subsequent open-ended question. Those who stated “no, the PHS is not attractive” were asked why they did not consider the PHS as attractive. Both groups were also asked to provide suggestions on what would need to change in order to consider the PHS as (more) attractive. Finally, all participants had the opportunity to answer a further open-ended question at the end of the survey, giving them the opportunity to provide any further comments regarding the survey and/or the PHS.

After project completion, the quantitative data of The OeGD-Studisurvey will be available on https://osf.io/uxftz (access on 5 September 2022). To prevent identification of individuals through the combination of the open-ended answers and socio-demographic data, the unredacted dataset will not be publicly available due to privacy reasons. The data presented in this study are available on request from the corresponding author.

### 2.3. Coding of Textual Elements Including Qualitative and Quantitative Content Analysis

The written statements were analyzed using content analysis, following the approach outlined by Mayring [30,31]. First, a coding frame was developed in a mixed deductive and inductive approach: a preliminary coding frame was created inductively (J.M.S) based on coding a sample of responses, while keeping the logic model presented in the companioning paper [29], previous brainstorming within the group, as well as the relevant literature on the topic in mind. The coding frame consists of a broad number of specific themes (level 3 codes), which were further subsumed in smaller aggregated units addressing the same or a similar main theme (level 2 codes) and finally grouped into a small number of overarching themes (level 1 codes). This preliminary coding frame was discussed and revised, at first through discussion with a second researcher (L.A.) and in a next step with the larger research group. The revised coding frame was then applied by one author (J.M.S.) and—to ensure intersubjectivity in the application of codes—checked by at least two other authors (L.A., F.F., F.H., L.K., L.J., A.M., M.R.), with conflicts being resolved in discussion between two authors (L.A., J.M.S.).

Prior to the coding of the responses and to maximize intersubjectivity, consistency and reproducibility, we developed a coding guidance consisting of a brief summary of what the particular code refers to as well as specific characteristics of the textual element or keywords in the text to which this code should be applied or not be applied. The coding frame can be downloaded at Supplement Material Table S1, the coding guidance is provided upon request.

The coding process was conducted in an excel spreadsheet (Microsoft Excel, 2013). All individuals who provided one or more responses to the perceived attractiveness of PHS were included in the analysis. This involved assigning one or more codes to individual participants (represented by a row in the data analysis document) that represented a participants’ reasons for (not) considering the PHS attractive across all responses to the respective question. In a next stage, two authors (J.M.S., L.A.) revised each code (level 3 codes) including the linked responses and assessed its content and relation to other codes to reassess and reorder the grouping of codes into the two levels of clusters (level 2 and level 1 codes). This was carried out to group codes with a similar or closely linked theme into a smaller set of level 2 codes. Within this process, the authors also summarized and synthesized key themes, including contextualization and relation to other codes. Two authors (J.M.S., L.A.) selected exemplary quotes that best highlighted the themes (or particular aspects of it) in close discussion. In the results section, we provide translated anchor examples for the respective codes. The original German version of all cited statements, as given in the survey, is provided in Table A7 in Appendix C.

In a final step, one author (J.M.S.) conducted a descriptive statistical analysis through quantifying how many individuals made a statement to which a particular code was assigned. The analysis was stratified by the field of study (medicine vs. public health (PH) and other non-medical (ONM) studies of relevance for the PHS). Unadjusted comparisons were made between medical students and PH&ONM students using Pearson’s Chi² test (or Fisher’s exact test, as appropriate) for categorical variables, as well as between the responses of wave 1 and wave 2 for medical and PH&ONM students. In addition, we created a table depicting for all codes how often they were assigned to a statement together with any of the other codes used. This allowed us to determine, for example, how often statements coded with “the PHS being too bureaucratic” were also assigned to the code reflecting the reason of the “salary being too low”. These quantitative analyses were performed with R Studio version 2022.02 [32].

## 3. Results

### 3.1. Characteristics of the Study Population

Of the 1835 medical students in wave 1 and the 223 medical students in wave 2, 865 (47%) and 180 (81%), respectively, responded to the question on whether they considered the PHS to be attractive or unattractive for future employment. Among PH students and other ONM students, these figures were 45% for wave 1 and 76% for wave 2. Among those who answered these questions, the majority (91% of medical students and 84% of PH&ONM students across both waves) also provided a written statement and were thereby included in the analysis.

Key characteristics of the included study participants are provided in Table 1. While there were no differences in gender compared between wave 1 and 2, the study population in wave 1 was slightly younger than in wave 2. Fewer medical students participated in wave 2, in particular, fewer medical students without PHS interest. The proportion of individuals who stated that they could imagine working in the PHS was higher in wave 2—likely due to the lower participation rates of medical students in wave 2, as medical students stated at lower rates that they could imagine working in the PHS [29].

Among medical students providing a valid (i.e., written) response to the question, 30% across both waves stated that they considered the PHS as attractive, and 70% considered it not attractive. However, the population of those having selected the closed, binary response of considering the PHS as attractive or not attractive does not completely overlap with the population that presented arguments for or against the PHS being an attractive employer. Here, 72% of medical students brought forward at least one argument against the PHS being attractive to them, while 33% provided at least one argument in favor of the PHS. Among PH&ONM students, 63% rated the PHS as attractive, and 62% provided at least one argument in favor (two individuals stated that they had too little knowledge about the PHS). In addition, 37% considered the PHS as unattractive, but 45% of PH&ONM students provided at least one argument against the PHS being attractive to them as young professionals (Table 2).

In both waves of the survey, the participants were asked whether they could imagine working in the PHS. The responses to this question and factors associated with this response are presented in more detail in the companioning publication [29].

### 3.2. Reasons Why Working in the PHS Is Perceived as Attractive or Not Attractive

Across both waves, medical students who stated that they could imagine working in the PHS (“yes” or “rather yes”, in the following “with PHS interest”), 72% provided at least one argument why they considered the PHS as attractive, while 34% provided at least one argument why they did not. Among the medical students who could not imagine working in the PHS (“no” or “rather no”, in the following “without PHS interest”), 14% stated the PHS being attractive, and 90% considered PHS as not being attractive.

**Figure 1:**
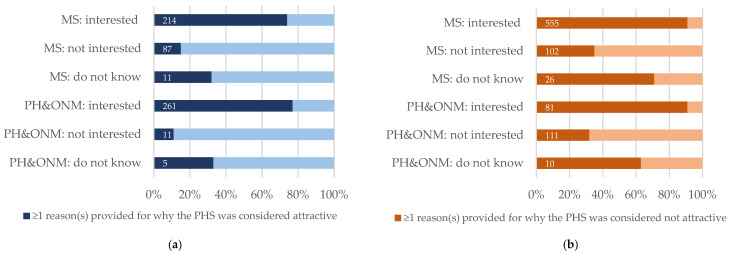
Interest in working in the PHS and perceived attractiveness of the PHS. Number and proportion of individuals (**a**) who provided at least one argument in favor of the PHS being an attractive employer and (**b**) who provided at least one argument against the PHS being an attractive employer by their response to the question of whether they could imagine working in the PHS and who could not imagine working in the PHS *(Abbreviations: MS: medical students; PH&ONM: public health and other non-medical students; PHS: Public Health Service)*.

In contrast, among PH&ONM students with PHS interest, 77% provided at least one reason for why they considered the PHS as attractive, while 33% made at least one reason for the perceived unattractiveness of the PHS. Among those PH&ONM students who could not imagine working in the PHS, 90% provided at least one argument against the PHS being attractive, while 12% brought forward at least one argument in favor of the attractiveness of the PHS (Figure 1).

#### 3.2.1. Main Reasons for Why Working in the PHS Was Not Considered as Attractive

Table 3 provides an overview of the main reasons provided for the PHS not being considered attractive as well as those codes classified as neutral (e.g., lack of knowledge about the PHS), on the level of the aggregated codes (level 1 and level 2 codes). More comprehensive versions of Table 3, including level 3 codes that were provided by more than five people and tests for significance between medical and PH&ONM as well as between wave 1 and wave 2, can be found in Table A1 (extended table), Table A3 and Table A4 (disaggregated by wave 1 and wave 2) in the Appendix B.

Overall, a broad range of different reasons were provided, with considerable differences within as well as between the groups of medical and PH&ONM students. In total, 683 medical and 202 PH&ONM students provided at least one argument against the PHS being attractive, at least one statement related to the lack of knowledge or provided an unclear or ambiguous response. In the following, we refer to this subgroup when writing about the proportion of medical or PH&ONM students providing a reason or motive, unless otherwise specified.

Across both waves, 19% of medical and 17% of PH&ONM students who provided arguments against the PHS being attractive stated they were (categorically) not interested in working in the PHS or had other professional interests incompatible with working in the PHS. Among medical students, the main reasons for considering the PHS as not attractive were (i) the work not containing enough (clinical) medicine (57%), (ii) the occupation in the PHS or the PHS itself being perceived as too bureaucratic (25%), (iii) the occupation in the PHS being considered as too boring, monotonous, or generally not interesting (12%), (iv) the salary and remuneration being too low (12%), and (v) the PHS being experienced as outdated, not innovative, or in need of modernization (8%).

Among PH&ONM students, the main reasons for considering the PHS as not attractive were (i) the occupation in the PHS or the PHS as an institution being considered as too bureaucratic (37%), (ii) the PHS being perceived as outdated, not innovative, and/or in need of modernization (28%), (iii) a lack of creative freedoms in the occupation (15%), (iv) the PHS not providing job opportunities for and/or sufficient acknowledgement of individuals without a medical degree (10%), and (v) the salary and remuneration being considered as too low (9%).

#### 3.2.2. Insufficient Knowledge

In total, 23% of the medical and 17% of the PH&ONM students stated they knew little about the PHS. Repeatedly, these statements were made in the context of having too little knowledge to hold an established position for or against working in the PHS. However, with 98% of medical and 100% of PH&ONM students, the vast majority of those having made this statement declared that they did not consider the PHS to be an attractive employer. For a minority, it was the only argument put forward against (or in favor) of the PHS being attractive. Most combined this statement with other reasons against working in the PHS, such as the PHS being too bureaucratic. Both medical and PH&ONM students suggested that providing internships (including medical internships such as the *Famulatur* or the practical year) or improving the presence of the PHS in the study curriculum could increase the knowledge and thereby the attractiveness of the PHS. In the following, we provide exemplary responses to highlight these themes.
*“[The PHS is not considered attractive, as] it is unclear what the PHS offers [and] which areas of work working there would entail. The transparency is missing and it is not clear to me, to what extent an active career working with patient would be possible on a fixed position in the PHS”**(W1. ID467)**“[The PHS is not considered attractive, as it is] remote from patients (hardly any direct, longer-lasting, therapeutic patient contact); bureaucracy (I guess- actually had no insight so far)”**(W1. ID496)**“So far, I have hardly had any insight, therefore: [in order to become more attractive, the PHS should] become present in the first place, become a topic in the curricula, present itself in an interesting way”**(W1. ID710)**“I think, [to increase the attractiveness of the PHS] there should be more reporting on the practical tasks of the PHS. I can’t imagine any concrete activities under ‹‹hygiene management›› and ‹‹drinking water monitoring››-and the generic terms sound rather boring to me as a future job”**(W1. ID856)*

#### 3.2.3. Categorical Lack of Interest in Working in the PHS or No Suggestion on How to Increase the Attractiveness of the PHS

In total, 19% of medical and 17% of PH&ONM students stated that they were generally or categorically not interested in working in the PHS:
*“[The PHS] does not necessarily correspond to my interests”**(W1. ID910)*

Additionally, a further 29% of medical and 17% of PH&ONM students provided one or more reasons for why they considered the PHS as not attractive but did not provide any suggestion on how to increase the attractiveness of the PHS. Among all respondents falling into one of these two categories, 51% (n = 207) stated that they did not consider the PHS as attractive due to the lack of clinical/patient-centered work or because they were interested in pursuing a career path in clinical/patient-centered work. Slightly more than 8% (n = 33) of those providing either of the two responses indicated that they could not provide any suggestions on how to make the PHS more attractive, as they knew too little about the PHS:
*“[The PHS is not attractive for employment, as] I am pursuing other specific professional goals and these do not fit with a job in the PHS”**(W1. ID878)**“[The PHS is not attractive for employment, as] I would like to go in the direction of research and development of pharmaceuticals. [For the PHS to become more attractive], my goals for career choices would need to change”**(W1. ID1016)*

The most prominent reason among medical students for why the PHS is not attractive was that the work entailed too little clinical practice and/or too little contact with patients. This was stated by 57% of medical and 5% of PH&ONM students, namely individuals with a background in physiology, psychology, or nursing. This cluster of codes included an explicit statement of the work entailing too little clinical practice and/or too little contact with patients (stated by 54% of medical students), statements that in order to become attractive, the medical component of the work would need to be increased (10% of medical students), as well as statements that the work at the PHS would be too focused on public health and/or prevention (2% of medical students). Most often, arguments of insufficient clinical practice and/or contact to patients were made in combination with a statement of categorically not being interested in working in the PHS or not making any suggestion for how the PHS could be made more attractive. Others contrasted the practical work in the hospital or medical practice with the characteristics bureaucratic or theoretical ascribed to the work conducted in the PHS.
*“[The PHS is not attractive for employment,] because I would like to work primarily as a practicing physician with ill patients in the clinic. [...] [For the PHS to become more attractive to me, the] concrete patient treatment would always have to be my main task”**(W1. ID447)**“[The PHS is not attractive to me, as] I think that the work does not include enough medical practice [...]” [Response to the question of how the PHS can become more attractive?] Nothing, really. The image that I have of the PHS contains little clinical practice, and since I want to become a physician, this career field is rather not for me”**(W1. ID774)*

#### 3.2.4. Too Bureaucratic Work and Institutions

The PHS being too bureaucratic was a prominent reason for not considering the PHS as attractive and was stated by 25% of all medical and 37% of all PH&ONM students. One set of arguments within this cluster of codes focused on the work within the PHS being bureaucratic in the colloquial sense of the word, i.e., tasks characterized by excessive documentation, administration, and similar “paperwork”. Here, bureaucratic office and paperwork was repeatedly contrasted with “practical” (often clinical) work. This perception was more prominent among medical than among PH&ONM students:
*“[The PHS is not attractive to me, as] in the PHS you only do administrative tasks and the people who work in the PHS are very dissatisfied”**(W1. ID549)*

A second set of reasons for considering the PHS as unattractive within this cluster characterized the PHS as a bureaucratic institution, i.e., being an institution characterized by impersonal administrators, strict adherence to fixed rules and regulations, and hierarchies of authorities and responsibilities. The PHS was repeatedly characterized as an institution bound and restricted by rules, regulations, and its bureaucratic nature in general.
*“[The PHS is not attractive to me, due to] too much bureaucracy and strong hierarchy (little opportunity to contribute own ideas), effects/successes are not directly apparent”**(W1. ID405)**“[The PHS is not attractive to me, as] in my opinion, there are too many regulations and laws in combination with bureaucracy that you have to solve. In addition, I would then also have to take on many administrative tasks, which is rather uninteresting for me”**(W1. ID918)*

Hence, the PHS was characterized as too rigid, inflexible as well as not being dynamic or being too slow to act. In this context, when students stated that the PHS would have no or only limited impact (4% of medical and 6% of PH&ONM students) or would not allow for adequate creative freedoms (5% of medical and 15% of PH&ONM students), it was often related to the institution as well as to the professionals in the institution being impeded by this bureaucratic nature, which was characterized as being too frustrating by some.
*“[The PHS is not attractive to me, as] the bureaucracy works too inefficiently and too slowly to really change/shape anything”**(W1. ID964)**“[The PHS is not attractive to me, as there are] too few real successes, due to high bureaucracy etc; [The] feeling that nothing can be achieved due to numerous regulations”**(W1. ID390)*

Within this context, respondents repeatedly alluded to negative stereotypes of public authorities (in German: Behörden) or civil servants (in German: Beamte) common in Germany, e.g., individuals working in the PHS being primarily interested in working as little as possible. These statements were repeatedly linked with the reason provided by 4% of medical and PH&ONM students for why the PHS was not attractive to them, namely it not being challenging enough and/or being unattractive for people with ambitions in general.
*“[The PHS is not attractive to me, as] I don’t want to sit in the office, write expert reports [in German: Gutachten] and only discuss responsibilities (‹‹that’s another person’s responsibility›› or ‹‹the main goal is as little work as possible››)”**(W1. ID645)*

#### 3.2.5. Outdated, Not Innovative, and in Need for Modernization

A major reason for the PHS being considered as unattractive among PH&ONM students (28%) as well as among medical students (8%) was that the PHS was outdated, not innovative, and in need of modernization. Most of the time, this code was applied to statements on how the PHS could become more attractive: Here, participants repeatedly stated that the PHS would need to become more modern, more innovative, more digitalized, and/or statements calling for a general need for reform. While most of the statements were general (e.g., general calls for the PHS to become more modern), others highlighted specific aspects where they regarded improvement or modernization to be necessary, such as regarding administration, organization of work and working culture (e.g., becoming more similar to the work in start-up companies; as suggested by a participant), or that more modern and innovative public health approaches would be necessary.
*“[In order to become more attractive, the PHS would need to] be innovative, modern, flexible and, above all, unbureaucratic”**(W1. ID865)**“[In order to become more attractive, the PHS would need to] strengthen digitalization and efficient implementation of new ideas”**(W2. ID197)**“Germany is unattractive because [it is] extremely lagging behind and a structural catastrophe in the areas of public and global health, [in the field of] innovation in the health sector/system, [and in the field of] transdisciplinary research”**(W1. ID968)*

#### 3.2.6. Occupation Lacking Creative Freedoms

A further prominent theme was the lack of creative freedoms (in German: Gestaltungsspielraum) associated with an occupation in the PHS; primarily among PH&ONM students (15%) but also among medical students (5%). These arguments referred to lack of creative freedoms, in general or aspects such as, of room for developing and following up on own ideas, of developing own projects, and of freedoms in making decisions, as well as calls that this would need to be provided in order for the PHS to become attractive. Mostly, these statements were made in the context of the PHS being too bureaucratic, i.e., that the rules, regulations, or hierarchies would limit the individual creative freedoms.
*“[The PHS is not an attractive employer, because] I want to contribute my own ideas and conceptions to work more independently of public regulations and instructions of employers”**(W1. ID624)**“[The PHS is not an attractive employer, because of] too much bureaucracy and strong hierarchy (little opportunity to contribute own ideas)”**(W1. ID405)*

#### 3.2.7. PHS Not Providing Job Opportunities for and/or Sufficient Acknowledgement of Individuals without a Medical Degree

A prominent cluster among PH&ONM students (10%) (but not among medical students (<1%)) was that jobs in the PHS were not available or were at least not sufficiently accessible for individuals without a medical degree, as well as that these non-medical professionals would not receive sufficient acknowledgement within the PHS. Among those providing these reasons, it repeatedly was the only reason against the PHS being attractive and/or suggestions for how the PHS could become more attractive. These statements were made both by individuals with a background in public health, as well as by those with other professional backgrounds, such as nursing.
*“[In order to become more attractive] the PHS needs more space for non-medical staff”**(W2. ID141)**“[The PHS is not an attractive employer, as] there are no jobs for non-physicians”**(W2. ID353)*

#### 3.2.8. Salary and Remuneration Are Too Low

A further reason regarding job and employment, which was brought forward by 12% of medical and 9% of PH&ONM students, was that the salary and remuneration was perceived as being too low. Here, most medical students compared the salary within the PHS with the salary to be expected when working in a hospital or in private practice, to the detriment of the PHS. Repeatedly but often in combination with other suggestions on how to improve the PHS, it was stated that to become more attractive, the salary of individuals with a medical degree would need to be aligned with the salary expected by physicians working in hospitals and private practices. Some of those explicitly emphasized the high levels of professional qualification of medical specialists, partly by contrasting the salary for physicians with the salary for other professions perceived as less professionally qualified. Another aspect, often brought forward in this context, was that working in the PHS came with a tradeoff: having a relatively relaxed job with good working hours but having to live with a lower salary. A smaller subgroup criticized that the remuneration was not performance oriented, i.e., hard and intensive work would not lead to a higher salary, making the occupation unattractive for individuals with a strong career orientation.
*“[The PHS is not attractive because] you can’t achieve anything that is not politically opportune, and the payment does not in the slightest reflect your own qualifications (expecting to recruit a medical specialist with a salary that is less or at most that of a high school teacher is simply laughable)”**(W1. ID918)**“[The PHS is not attractive because] the remuneration and appreciation of [medical] doctors in the PHS are completely below average. […]. [In order to become more attractive], definitely, there would have to be a doctors’ tariff within the PHS, cancelling out the differences in remuneration compared to clinicians working in hospitals”**(W1. ID956)*

#### 3.2.9. Occupation Being Boring, Monotonous, or Generally Not Being Interesting

That the occupation in the PHS was regarded as boring, monotonous, and/or generally not interesting was brought forward as a reason for the perceived lack of attractiveness by 12% of medical and 7% of PH&OMS. Often, no further explanation beyond the statement that the expected occupation being boring, repetitive, or monotonous was provided. Others provided more contextual information and ascribed the perceived boredom and repetitiveness to the aforementioned bureaucracy or bureaucratic tasks (“boring office work”), to the lack of clinical practical work (primarily among medical students), and the work generally not being demanding or challenging.
*“[In order for the PHS to become attractive] it would have to prove to me that the PHS has a colorful range of work fields, promotes innovative and active research and actually influences politics. One could also say that the PHS should show that it does not only consist of dull, dry and inefficient bureaucratic processes and that the only desirable thing is the pension afterwards”**(W1. ID940)**“I may also be too little informed. But [I do not consider the PHS as attractive because] above all I associate monotonous activities and the work of public health officers [in German: Amtsarzt] (ergo not helping (?)) activities with it.”**(W1. ID622)*

### 3.3. Main Reasons Provided for Why Working in the PHS Was Considered Attractive

Among those providing a valid written response, 62% of PH&ONM students (n = 278) and 33% of medical students (n = 312) provided at least one argument regarding what makes the PHS attractive to them. Again, as with the reasons provided against the PHS being attractive, this subpopulation was used as a reference group when referring to the proportion of individuals providing a reason for why they considered the PHS as attractive. As with the main reasons for the PHS being considered as unattractive, the reasons provided varied. While the five most prominent reasons among medical and PH&ONM students were identical, they were cited to varying degrees (Table 4). An expanded version can be found in Table A2 and Table A4 in Appendix B.

Among medical students, the main provided reasons for considering the PHS as attractive were (i) a good work-life balance and attractive working hours (51%), (ii) that the tasks and activities of the PHS involve or are focused on action on the population level, addressing underlying structures or systems, such as social determinants of health (SDH), or prevention (21%), (iii) having impact or making a difference (17%), (iv) the occupation being exciting, diverse, or generally interesting (17%), and a high degree of job security (13%).

Among PH&ONM students, the most prominent arguments were (i) the PHS offering a high degree of job security (28%), (ii) that the tasks and activities of the PHS involve or are focused on action on the population level, addressing underlying structures or systems (SDH), or prevention (24%), (iii) the occupation being exciting, diverse, or generally interesting (23%), (iv) having impact or making a difference (23%), (v) good work-life balance and attractive working hours (14%).

#### 3.3.1. Good Work-Life Balance and Attractive Working hours

The reason of the PHS being attractive, due to providing a good work-life balance or attractive working hours or working-time models was also repeatedly stated, in total by 51% of medical and 14% PH&ONM students. This included the perception of predictable working hours (22% of medical and 3% of PH&ONM students), the PHS being a family friendly employer and/or providing family friendly working hours (15% of medical and 4% of PH&ONM students) and providing a good work-life balance (10% of medical and 6% of PH&ONM students). Furthermore, 8% and 7% of medical students, respectively, stated that the lack of shift work and lack of working at nights or on weekends, as well as the length of working days and hours, were considered attractive. These arguments were provided by less than 1% of PH&ONM students.

Repeatedly, these arguments were made in the context of the job being relaxed and the overall workload being low, arguments provided by in total 7% of medical and 4% of PH&ONM students. Among the majority of individuals providing this reason, statements to which codes within the cluster addressing a good work-life balance and attractive working hours were the only reasons provided:
*“[The PHS is attractive to me, because of] family-friendly working hours (models), no weekend or night shifts”**(W2. ID155)**“[The PHS is attractive to me, because there] family is probably more compatible with the job than in shift work in the hospital”**(W2. ID156)*

#### 3.3.2. Addressing Health Challenges on a Population Level, on a Systemic or Structural Level, or through Prevention

The third most prevalent reason provided by 21% of medical and 24% of PH&ONM students was that they considered the PHS as attractive, as it (or oneself when working within the PHS) would be able to address health challenges on a population level, address health challenges on a systemic or structural level, or through prevention. This includes 12% among medical and 15% among PH&ONM students who were interested in action on a population level, 8% among medical and 7% among PH&ONM students who referred in their reasoning to the systemic or structural dimension of health and well-being, as well as 8% of medical and 7% of PH&ONM students who referred to prevention in their reasoning.
*“[The PHS is attractive to me,] as I would like to work in prevention and not just do damage control in the other fields”**(W2. ID106)**“[The PHS is attractive to me, because] I want to improve the health of people at the population level in a preventive and health-promoting way”**(W2. ID118)*

A further 3% among medical and 5% among PH&ONM students who referred to the PHS as not being attractive indicated that they were interested in affecting population health on a structural or systemic level or in prevention; however, they considered that the PHS did not provide sufficient opportunities to do so or was not active enough in this regard.
*“[The PHS is not attractive to me, as] currently [it] does not promote and fulfill the kind of public health I want to be engaged in. The PHS does much more complementary [medically-oriented] health care and bureaucratic tasks than working innovatively to improve and promote population health and advocating for it at the political level”**(W1. ID973)*

#### 3.3.3. Having Impact or Making a Difference

A further prominent theme regarding the attractiveness of the PHS is having impact or making a difference, as stated by 17% of medical and 23% of PH&ONM students. This cluster of codes included the three major themes of in general having a positive impact or experiencing to make a difference, the theme of maximizing impact on health (e.g., through positively affecting many people), and the PHS being an important institution for promoting public health, as well as the minor theme of having a positive impact explicitly on vulnerable and marginalized individuals and communities. Repeatedly and in particular when referring to the theme of maximizing impact, the participants also referred to the PHS as being active on a population, rather than an individual level.
*“[The PHS is attractive to me, because there] I can actively do something for the health of larger groups of people & not only on an individual level”**(W2. ID130)**“[The PHS is attractive to me, because] at the end of the day you can go home with the feeling of having done something good”**(W2. ID77)**“[The PHS is attractive, as] it takes on important tasks for the society”**(W1. ID327)*

By contrast, a lack of impact or the expected lacking experience of making a difference was a reason brought forward by 4% of medical and 5% of public health students who provided at least one reason for why they considered the PHS as not attractive. Often, these statements were either made in the context of lacking clinical work (i.e., a physician helping a sick individual) or in the context of bureaucracy or politics limiting the ability of the PHS to act.
*“[The PHS is not attractive, because] I want to help people”**(W1. ID693)**“[In order to become attractive,] it would have to become more capable of acting and make me believe that necessary decisions can actually be made and implemented effectively”**(W1. ID718)*

#### 3.3.4. The Occupation Being Exciting, Diverse, or Generally Interesting

While the occupation in the PHS being considered as boring, monotonous, and repetitive was a prominent reason for not considering the PHS attractive, 17% of medical and 23% of PH&ONM students considered the PHS as attractive due to the occupation being interesting, diverse, or exciting. In this context, participants most often referred to the attractiveness of the broad scope of themes, topics, and activities within the PHS, while others described the occupation as generally interesting, or expressed an interest in specific PHS related topics (e.g., infectious disease prevention, health reporting).
*“[The PHS is attractive, as] interesting, varied work with a wide reach”**(W2. ID86)**“[The PHS is attractive, as] it offers a wide range of projects in which you can get involved and thus make the world a little bit better”**(W1. ID291)**“[The PHS is attractive, as] I would expect the work as not being limited buntly to one’s own subject but to take on various tasks”**(W2. ID121)*

#### 3.3.5. High Degree of Job Security

Finally, the PHS providing an occupation with a high level of job security was provided as a main reason for considering the PHS attractive by 28% of PH&ONM students, and to a lesser extent by medical students (13%). Repeatedly, this reason was provided in combination with references to attractive work-life balances and the family friendliness of an employment in the PHS.
*“[The PHS is attractive, because it provides] security with regard to job guarantee and contract duration, [as well as because of the] salary structure, working hours, [and] relevance of the job”**(W2. ID34)*

### 3.4. Previous Experience

In wave 2, a total of 29% of medical and 50% of PH&ONM students included in this analysis reported having experience with working in the PHS (e.g., through internships, work, conferences or projects). Among those, 48% of medical and 70% of PH&ONM students provided at least one reason for why they considered the PHS attractive, in contrast to 38% of medical and 60% of PH&ONM students who reported not to have practical experience in the PHS. Regarding statements on why they considered the PHS as not attractive, 58% of medical and 40% of PH&ONM students with experience provided at least one argument, in contrast to 66% of medical students and 51% of PH&ONM students without experience. However, these differences were not significant (Table A5 in Appendix B).

Among medical students with previous PHS experience, the main reasons for considering the PHS as not attractive were (i) the work not containing enough (clinical) medicine (39%), (ii) the occupation in the PHS or the PHS itself being considered too bureaucratic (36%), (iii) the occupation in the PHS being considered as too boring, monotonous, or generally not interesting (11%), (iv) the salary and remuneration being too low (11%), and (v) the occupation providing limited creative freedoms (11%) (Table A6 in Appendix B). Among PH&ONM students with previous PHS experience, the main reasons for considering the PHS as not attractive were (i) the PHS being considered as outdated, not innovative, and/or in need of modernization (36%), (ii) the occupation in the PHS or the PHS as an institution being considered as too bureaucratic (29%), (iii) the PHS not providing job opportunities for and/or sufficient acknowledgement of individuals without a medical degree (12%), (iv) the salary and remuneration being considered as too low, and (v) the occupation offering too little creative freedoms.

Thereby, the main reasons for not considering the PHS as attractive among students with PHS experience were the same and appeared in the same order of importance as in the overall study population. Furthermore, among those responding to the questions, several referred to their experience in the PHS and explicitly stated that having experiences in the PHS had not increased the perceived attractiveness of the PHS, e.g.,
*“I worked in a local health authority (LHA) during the pandemic in 2020–2021 and was very disappointed by the entrenched structure and the ‹‹stuckness›› of the employees. I hope that with a generational change, more openness and motivation will come to the LHA. Unfortunately, there was no planning ahead. Motivated and very well-qualified students had no chance for a (long-term) employment above pay group 4 (with master’s degree) and we all looked for other employers”**(W2. ID165)**“I had only a vague idea of the work of the LHA before the pandemic (Just with the stereotype of a job where no one overworks themselves). But after one year of the pandemic and working in the LHA, I see this prejudice not only confirmed, but exceeded to a great extent. I am shocked, disappointed and stunned by the overall performance of the PHS during the pandemic”**(W2. ID114)*

## 4. Discussion

Among medical, 33%, and among PH&ONM students, 62% provided at least one argument for why they considered the PHS as attractive, while 72% of medical and 45% of PH&ONM students provided at least one reason for why they did not consider it as attractive. These figures are largely overlapping with the proportion of individuals who stated that they could imagine working in the PHS, as found in the quantitative analysis of the OeGD-Studisurvey [29] and the Berufsmonitoring Medizinstudierende 2018, a large survey of medical students conducted in 2018 [10] (with 29% and 23% of the more than 13.000 participants, respectively).

As proposed by experts and stakeholders [5,11,12,13], focusing on those characteristics of the PHS regarded as positive, e.g., by emphasizing them in public awareness campaigns or by further expanding on them when reforming the PHS, could be an effective strategy in increasing the attractiveness of the PHS among young professionals.

However, a high number of individuals provided at least one reason for why they did not consider the PHS as attractive, and the finding that around one in three participants who could imagine working in the PHS provided at least one reason for why they did not consider it attractive should give rise to concern. This aligns with statements by young professionals who would like to work in public health, but emphasize the need for reform in the PHS to become an attractive option for young professionals [13,19]. If a sustainable recruitment and retention of young professionals in the public health workforce in Germany is to be achieved, the concerns raised should be taken seriously.

While several themes were brought up by the participants, they considered the PHS as (not) attractive for different reasons. Therefore, the attempts to increase the attractiveness of the PHS will likely need to reflect his heterogeneity and a one-size-fits-all solution will probably not be able to achieve the intended impact. This may include messages targeted and tailored to specific groups as well as addressing different targets of reform of the PHS. Due to the heterogeneity in reasons provided within this survey, the messaging to attract young professionals with and without a medical degree would need to be different as well.


***Key finding 1.** Some individuals are simply not interested—trying to convince everyone is likely not the most efficient use of limited resources*


Around one in five participants explicitly stated that they were categorically not interested in working in the PHS, for example, they had a different career path in mind. This argument was often provided in conjunction with the intention to work in the curative health care sector, and was by far the most prominent aspect among medical students for considering the PHS as unattractive. This was reflected in the quantitative analysis of the OeGD-Studisurvey [29] as well as in the Berufsmonitoring Medizinstudierende 2018 [10] where more than nine out of ten medical students stated that their primary career focus lay on working in hospitals or in private medical practice.

The attractiveness of a job, i.e., how satisfied an individual (expects) to be in an occupation is affected by many different factors, such as interest in the tasks to be performed, the amount of pay, or the perceived significance of the tasks [33]. According to Locke’s Range of Affect Theory, (expected) job dissatisfaction results from a job not providing or not being expected to provide a job facet valued by a person, while (expected) job satisfaction arises when those expectations are met [33]. The theory furthermore states that individuals value facets of work differently — leading to differences in (expected) job (dis-)satisfaction within the same occupation [33]. Thus, if a person clearly and categorically rules out an interest in working in the PHS, this could be due to the facets of work provided within the PHS that do not match that person’s values and preferences, or because other occupations are known or expected to better match those expectations.

Therefore, in light of limited resources available and constraints on the political capital needed for reform, the following should be considered: convincing individuals who categorically rule out working in the PHS will likely require much more resources and more fundamental institutional changes, as would overcoming the barriers perceived by those who already regard the PHS as attractive to some extent.


***Key finding 2.** Overcoming a lack of knowledge about the importance of the PHS*


One prominent theme was statements from participants (both medical and PH&ONM students) who stated to know little about the PHS. These statements match findings for example from the Berufsmonitoring Medizinstudierende 2018, where only 4.5% of all surveyed medical students considered themselves well informed about occupation and working conditions within the PHS [10]. We are not aware of similar surveys among PH&ONM students.

In light of Locke’s Range of Affect Theory [33], overcoming these gaps of knowledge may increase the perceived attractiveness of the PHS through increasing expected job satisfaction: Not being aware that facets of work valued by an individual may match with the facets of work provided in an occupation within the PHS could impede individuals in engaging in a career path within the PHS.

Increasing exposure to the PHS by anchoring PHS-related topics into the curriculum and through practical insights is among the approaches most often discussed and proposed to enhance the attractiveness of the PHS, although often with a focus on medical students [7,13,15,16]. To date, several measures were already taken in this regard addressing medical students. For example, since the amendment of the medical licensing regulations for medical (in German: Approbationsordnung für Ärzte) was introduced in 2021, medical students now have the opportunity to complete a term of their obligatory medical electives (in German: Famulatur) and the final practical year (in German: Praktisches Jahr) within the PHS [34]. This is in line with other findings in our study, as statements about a lack of knowledge were often combined with suggestions to increase the presence of PHS-related topics in the curriculum or suggestions to allow for opportunities to gain insight into the PHS (e.g., through internships). The quantitative analysis of the OeGD-Studisurvey reported similar findings, where two out of three participants could imagine gaining some form of insight into the PHS during their studies [29].

Our findings indicate that more presence of the PHS during the course of study could help to overcome the lack of knowledge, which seems to be a barrier for medical as well as for non-medical students to get to know the PHS.


***Key finding 3.** Without structural reforms within the PHS, increasing exposure to the PHS will likely not have a major impact on the perceived attractiveness of the PHS*


However, our analysis indicates that increasing exposure to the PHS by itself is unlikely to be sufficient: When comparing the self-reported reasons provided by participants with and without previous experience in the PHS, both groups brought up similar reasons for not considering the PHS as attractive, even in the same order of importance. Furthermore, several participants explicitly referred to their experience in the PHS and stated that this did not lead them to consider the PHS as more attractive, but rather had the opposite effect. The quantitative analysis of the OeGD-Studisurvey showed similar findings: participants with and without experience had the same perception of the PHS across all but one thematic domain, such as the proportion of participants who considered the work in the PHS as not very challenging or demanding, as having little impact on population health, as being outdated and not innovative, or that working in the PHS was attractive for those people who are looking for a relaxed job [29].

The similarity in the perception of the PHS among participants with and without practical insights and experience indicates that the reasons brought forward by the students may at least partially be based on structural issues (i.e., reflecting real-world conditions), rather than them solely being unjustified (i.e., not true) negative prejudices. Hence, a one-sided focus on attempting to change the public image of the PHS (e.g., through large-scale image campaigns) or trying to increase exposure to the PHS (e.g., through electives or practical inside during studies) will likely not lead to a sustainable retention of young professionals, unless it is accompanied by structural reforms of the underlying issues — as those outlined in the following key findings.


***Key finding 4.** Emphasizing a good work-life balance in the PHS may increase the attractiveness of the PHS, but affirming existing negative stereotypes needs to be avoided*


The most prominent themes among medical students for considering the PHS as attractive were good work-life balance and attractive working hours associated with it (51% of medical students), which was also — although to a lesser extent — provided as a reason by roughly one in ten PH&ONM students. This positive image might be a valuable resource in attracting students and young professionals and could be utilized, for example, by emphasizing it in public awareness campaigns. In particular, emphasizing the perception of the PHS offering an overall good work-life balance, family friendliness, predictable working hours, and lack of shift work and less stressful working conditions compared to working in a hospital could be a helpful approach. This is also supported by the quantitative findings of the OeGD-Studisurvey, which found that good work-life balance was rated as most important among all work-related factors [29].

Therefore, the association of the PHS with comparatively good work-life balance and family friendliness could be helpful employer branding. However, in pursuing such a strategy, careful messaging is important: For one, this kind of messaging could play into and further emphasize existing stereotypes of the PHS being the ideal workplace for individuals seeking a job with low workload and is not attractive to individuals with goals and ambitions, a perception affirmed by three out of four medical students and every second PH&ONM student in the quantitative analysis of this survey [29]. Unintentionally reaffirming such stereotypes could reduce the attractiveness of the PHS, for example, among career-oriented individuals or those striving for high public health impact. Second, if not carefully worded, campaigns embracing primarily the message of a good work-life balance in the PHS could be perceived as alienating by those individuals who experienced a high workload in stressful environments in particular during the COVID-19-pandemic [27]. This may lead to the perception that their sacrifices were neither seen nor acknowledged. Due to its local anchoring, the public health service and, above all, the local health authorities are responsible for the prompt implementation of complex measures in times of crises. This requires enormous efforts and appropriate competencies to adapt to the new requirements within a short time. These challenges cannot be overshadowed by “simple” family-friendly employability branding.


***Key finding 5.** Emphasize and further strengthen the focus on population health, health promotion, and social determinants of health*


The focus of the PHS on population health, health promotion, or social determinants of health (SDH) was a prominent reason for considering the PHS as attractive, as was the reason of having impact, with these two themes often linked by the participants. This is reflected in the quantitative analysis of the OeGD-Studisurvey, where individuals who associate the PHS with low impact were less likely to be interested in the PHS [29].

We believe these findings are helpful to be interpreted in light of the Job Characteristics Model [35], a theory on job satisfaction with strong empirical foundation [36,37,38,39]. The model postulates that five key job characteristics influence critical psychological states that can directly affect work outcomes. According to the model, the key job characteristics skill variety (variety in tasks and skills needed to complete them), task identity (being involved in the entire process of a workpiece with a visible outcome, rather than only part of the work), and task significance (associating a sense of meaning with the task, such as having a tangible, positive effect) influence the critical psychological state of meaningfulness of the work. The job characteristic of autonomy can influence the experience of responsibility of outcomes, and the job characteristic of feedback mechanisms can influence one’s knowledge of the results. Together, all three critical psychological states then can lead to a number of potential outcomes, including (expected) job satisfaction [35].

A job characteristic of particular significance in this context is task significance, which refers to the sense of meaning associated with the task, such as having a tangible, positive effect on the world and other people. While having impact and making a difference was a prominent reason among participants considering the PHS as attractive, for others, the lack thereof was a reason why they considered the PHS unattractive. This may be in part due to participants overestimating the impact of medical approaches and innovations regarding its beneficial impact on health (i.e., a significant task), while underestimating the impact of non-pharmacological public health measures or addressing SDH, a prevalent misconception that we found among the participants within the quantitative analysis of the OeGD-Studisurvey [29] and which was also already found in the past [40]. A limited focus on the importance of public health and related themes during the medical studies (e.g., of SDHs [15,16]) may contribute to this issue. Hence, improving knowledge of the importance and the impact of public health action may increase the perceived attractiveness of the PHS by increasing the expected task significance and the expected meaningfulness of the work resulting from it.

In this context and given the prominence of wanting to focus on population health, health promotion, or social determinants of health (SDH) as a reason for considering the PHS as attractive, emphasizing these aspects and putting them in the center of public health awareness campaigns can be a successful strategy.

Focusing on this might be of particular importance, as health promotion and disease prevention (including a focus on vulnerable and marginalized individuals or addressing SDHs) were not consistently associated with the PHS. Within the survey, one out of four medical students and two out of three PH&ONM students expressed an interest in behavioral and primordial prevention (in German: Verhältnisprävention). However, out of those, roughly one in four did not consider this as a key function of the German PHS. Experts and stakeholders have criticized an insufficient focus in the PHS on these topics [41] and have emphasized the need to strengthen health promotion and disease prevention activities in Germany [13,42,43] and beyond [44]. Thus, our findings indicate that doing so could increase the perceived attractiveness of the PHS among young professionals.


***Key finding 6.** Reform bureaucratic structures and expansion of digitalization, but also empowerment of young professionals to act within the established administrative structures*


The most prominent reason for not considering the PHS as attractive among PH&ONM (37%) and to a lesser extent among medical students (25%) was that the PHS was regarded as too bureaucratic. This was often linked to the reasons of the PHS being regarded as outdated, not innovative or in need of modernization as well as that working within the PHS would not allow for creative freedoms. In this context, participants criticized both an overburden of administrative tasks as well as the adverse consequences resulting from them (e.g., slow decision making and action, limited creative freedoms). These reasons were repeatedly provided alongside with statements characterizing the occupation in the PHS as monotonous and boring. This was also seen in the quantitative analysis of the OeGD-Studisurvey, with nine out of ten participants considering the PHS as bureaucratic, and with less than one in five characterizing it as modern or innovative [29]. These perceptions were shared by both individuals with and without experience within the PHS [29]. Furthermore, similar findings have been reported on the satisfaction of the PHS in other countries [28].

Again, our findings are well reflected in the Job Characteristics Model [35], where a lack of core job dimensions is captured under what participants refer to or relate with bureaucracy: low skill variety (with working in the PHS is perceived as a monotonous office job in a public administration), low task significance (with extensive rules and regulations limiting swift and effective public health action), low autonomy (with hierarchical structures and regulation limiting the experience of responsibility for the outcome of the work), missing feedback and low task identify (with the compartmentalization of the work limiting the awareness about the results of the work).

Thus, a reform of the PHS toward a more modern public administration in which the core job dimensions are reflected seems to be necessary to increase attractiveness: streamlining overburdened administrative procedures, overhauling inefficient processes, expanding digitalization, and automating processes may considerably reduce the bureaucratic tasks considered as unattractive by students and young professionals. Expanding creative freedoms and an increasing responsiveness of the PHS (i.e., be more responsive to the needs of the community it serves) could also increase its attractiveness [45].

However, the PHS is part of the public administration and will always be bureaucratic to some extent, even after streamlining, digitalizing, or automating processes. Despite their negative image, bureaucracies themselves are not essentially problematic: in their ideal form, they were characterized as the most rational form of governance by the sociologist Max Weber, as they allow for predictability, consistency and protection from corruption [46]. Therefore, while the need for reform and modernization of overburdening bureaucracy within the PHS remains, both medical and PH&ONM students could benefit from more exposure to and training in public administration and sociology: First, this could raise awareness and appreciation of this rational form of governance. Second, the knowledge that the PHS always acts on behalf of the state and is therefore politically legitimized can contribute to a higher appreciation. Third, these skills could enable and empower young professionals to work within the system of public administration to implement change and improve health. 


***Key finding 7.** The salary gap needs addressing—but handled with care*


In our survey, roughly one in ten participants provided a low salary as a reason for not considering the PHS as attractive. These findings are in line with what we found in the quantitative analysis of the OeGD-Studisurvey, where receiving a high salary was regarded as important, but not among the top priorities for future working life among the participants [29]. This reason was less prominent than its importance in the public discourse suggested [1,8,11,12,13], with some experts also criticizing an overemphasis on the salary of PHS employees with medical degrees [41]. However, it needs to be kept in mind that the survey participants were students, whose lifestyle and salary expectations might change over time, including the importance attributed to the salary when making career choices. For example, other international studies have found dissatisfaction with the salary as a reason for employee dissatisfaction or intention to leave [25,28].

Regarding this reason, we believe Adams’ equity theory [47] provides a valuable frame of reference: it postulates that individuals expect compensation (e.g., salary, promotion, or recognition) that is fair in relation to what they are contributing (e.g., educational background, prior experience, or high job performance) as well as fair in relation to the compensation of their peers (i.e., their balance of what they contribute and receive). If the (expected) compensation is not perceived as in balance with their contribution (i.e., fair), this reduces their motivation and job satisfaction [47].

For example, in the OeGD-Studisurvey, medical students expressed that one of the reasons they did not regard the PHS as attractive was that their input, in the form of having a medical degree and an acquired a medical specialty, was not compensated fairly by the salary they would receive working in the PHS, in particular when compared to what their peers—physicians working in the health care sector—receive for the same input.

The problem of the existing salary gap between physicians in the PHS and in the health care sector needs to be addressed, if the PHS is to become more attractive to medical professionals. However, in light of other reasons for not considering the PHS an attractive employer, raising the salary without addressing other concerns is likely not the solution. Furthermore, this issue should be handled with care: reducing the salary gap between physicians within and outside of the PHS would lead to a further increase in the salary gap between medical and non-medical professionals within the PHS, which could spark social tensions. It may even reduce the attractiveness of the PHS among non-medical professionals, if this creates the perception that they and their work (i.e., their contribution) are not adequately valued and acknowledged, especially when compared to the input-output balance of medical professionals in the PHS [27,28].


***Key finding 8.** PH students perceive a lack of access to and acknowledgement in the PHS*


A final reason for not considering the PHS attractive, primarily among PH&ONM students, was that individuals without a medical degree would not receive adequate acknowledgement within the PHS or—despite interest—that the PHS would not provide adequate job opportunities for them.

This stands in contradiction to the high levels of interest in the PHS among PH&ONM students, found in both this analysis and the quantitative analysis of the OeGD Studisurvey [29]: Not only were considerably more PH&ONM students interested in working in the PHS as a career path, they were also more often interested in becoming a public health specialist (in German: Facharzt Öffentliches Gesundheitswesen), if Germany would implement something similar to the United Kingdom model [48], resulting in opening the public health specialist program to both individuals with and without a medical degree. However, simply opening up the residency training in public health to professional groups other than medical is not easily possible in Germany due to its strong medical tradition. Consideration should therefore be given to the extent to which career and qualification paths can be created for non-medical professionals that enable them to obtain certification analogous to the public health specialist in Germany (albeit in a different form). The academies for public health, as well as the chairs for PHS currently being planned at several universities in Germany [49], are likely to be important actors in order to ensure new and, above all, interdisciplinary training structures in the future.

Thus, our findings are consistent with the discourse led by experts and stakeholders calling for a reform of the PHS in Germany to create more open and flexible structures for young professionals without a medical degree [7,13,19,41]. Furthermore, they are in line with Adams’ equity theory [47]: if PH&ONM students believe that their qualification and training, their competences and knowledge, or their motivation will not receive fair compensation (e.g., in the form of acknowledgement), this may reduce the perceived attractiveness of the PHS and their motivation to seek a career within it. The findings also fit with the general discourse on strengthening the public health workforce in Germany, which largely focuses on medical professionals [1,8,41], although is not a discussion unique to Germany [45].

Individuals with a medical degree are essential for some core tasks within the PHS in Germany. However, currently, the potential of young professionals with high levels of interest to work for the PHS seems not to be adequately utilized. Strengthening the role of PH&ONM students within the PHS, reflecting on which skills and competencies are essential for which task as well as expanding the collaboration between disciplines and the appreciation for the expertise of every discipline contributing to the work of the PHS are likely crucial to sustainably strengthen the PH workforce in Germany.

## 5. Strengths and Limitations

Our study has several strengths and limitations. First, despite a comprehensive recruitment strategy, our sample is not a randomly selected sample. However, we found that the population of medical students in our survey, in particular in wave 1, was comparable to a large survey of more than 13,000 German medical students conducted in 2018 [10], for example regarding interest in working in the PHS or the interest in medical specializations. Unfortunately, we are not aware of such a document for PH&ONM students. It cannot be ruled out that through self-selection, individuals with interest in the PHS are overrepresented. However, due to our qualitative approach employed and due to conducting the analysis of reasons for considering and for not considering the PHS attractive separately, our main concern is representation rather than representativeness. In this regard, which we believe to have achieved, we found a saturation regarding both codes and meaning despite the heterogeneity of our sample.

Second, our analysis is based on the analysis of written responses to a survey. This approach does not allow one to further inquire to clarify ambiguous responses or explore complex topics in depth (e.g., understandings of bureaucracy). Here, additional research employing focus groups or key-informant interviews should be conducted.

Third, the population questioned in this survey comprises students, whose perception and attitudes might change in the early phase of the professional career. For example, medical students may overestimate the administrative work in the PHS and underestimate the administrative work in a clinical setting or change their attitudes toward the importance of salary. Here, conducting additional research among professionals in early stages of their career within and outside of the PHS might provide valuable insights.

Fourth, our survey was conducted before and in the early phases of the COVID-19 pandemic. During the pandemic, the PHS in Germany has received high levels of media, political, and public attention. This has likely raised awareness about the PHS among young professionals and may have sharpened their perception of it, both in a favorable or less favorable way. Additional research—for example in the form of a third wave of the OeGD Studisurvey—might be a solution.

Fifth, in order not to overwhelm the participants, we did not differentiate between the PHS on a local and on the federal or national level. As by far, most individuals working in the PHS are working on the local level, we believe this is the dominating frame of reference of the participants. This should be addressed in future publications.

Sixth, while we regard the qualitative approach in this publication to be a particular strength, further expanding on these findings through additional quantitative analysis (e.g., cluster and factor analyses) would provide additional insights. While this is beyond the scope of this publication, we aim to close this gap in an upcoming analysis.

However, despite these limitations, this survey—to the best of our knowledge—is the most comprehensive empirical study on the topic of young professional perceived attractiveness of the PHS in Germany conducted thus far. The overall lack of empirical evidence on the topic further emphasizes its importance. The qualitative approach utilized on responses by more than a thousand individuals allowed for an in-depth exploration of the diverse reasons for why the PHS was (not) considered attractive. Furthermore, the quantitative analysis of the qualitative data allowed for an estimation of the relative importance of these reasons. The approach of the OeGD-Studisurvey allowed us to interlink both qualitative and quantitative findings in a mixed-method approach.

## 6. Conclusions

Within this large-scale qualitative analysis of a survey among students, we identified a multiplicity of different reasons for why participants considered the PHS as attractive for future employment or did not do so. While the reasons for considering the PHS as attractive could be utilized for advertisement (e.g., in emphasizing them in public awareness campaigns), the reasons brought against the PHS should be taken seriously and should be addressed (e.g., by guiding reform in the PHS). Otherwise, a sustainable increase in attractiveness is likely not possible. Due to the multiplicity of reasons among participants, targeted and tailored approaches, rather than a one-size-fits-all solution, will probably be necessary to achieve the intended impact.

Given the clear and categorical rejection of the PHS by some parts of the population of interest, (i) utilizing limited resources on individuals with some inclination toward the PHS is likely more efficient, rather than indistinctly attempting to attract all (medical) students. Our findings indicate that (ii) increasing exposure to the PHS in the curriculum and through practical experiences could help to overcome barriers; however, (iii) if not accompanied with a reform addressing structural issues within the PHS, this will likely not be effective in the long term. (iv) The perception of the PHS offering a good work-life balance and family friendliness is a valuable resource for public awareness campaigns; however, overemphasizing this aspect could further strengthen negative stereotypes about the PHS and negate the immense management demands that are particularly common, although not exclusive, especially in times of crises. (v) By streamlining overly bureaucratic processes and modernizing the PHS overall as well as by (vi) strengthening the role of prevention and health promotion within the PHS, increasing knowledge about public health, and conveying the impact of the activities of the PHS on population health, the PHS could become more attractive to young professionals. While (vii) increasing salary was one reason for considering the PHS as not attractive, it was not the most central issue and needs to be handled with care to avoid social tensions within the PHS workforce. Finally, (viii) increasing the accessibility of the PHS to public health and other non-medical students and their acknowledgment and appreciation within it could allow the PHS to tap into a currently underutilized pool of resources to strengthen the PHS in Germany.

When addressing the role of PH&ONM professionals in the PHS as well as in reforming other areas of the PHS in Germany to increase job attractiveness, drawing on the practices and experiences of other countries in Europe and beyond can provide valuable insights [17,48,50,51,52,53,54]. Furthermore, such an endeavor is likely to benefit from a rigorous assessment of the situation in Germany, such as a field qualification analysis, mapping of public health training programs, as well as the systematic development of a catalogue of competencies for the PHS. 

## Figures and Tables

**Table 1:** Characteristics of the included study population of the OeGD-Studisurvey, total (n = 1393) and separated for wave 1 (n = 1021) and wave 2 (n = 372).

		Wave 1		Wave 2	
		(n)	(%)	(n)	(%)
Age	≤20	97	10%	36	10%
	21–25	535	52%	152	41%
	26–30	260	25%	73	20%
	>30	121	12%	62	17%
Gender	male	279	27%	96	26%
	female	737	72%	273	73%
	diverse/other	5	0%	3	1%
Field of study	medicine	720	71%	152	41%
	medicine in combination with another other non-medical study	65	6%	11	3%
	medicine and public health	18	2%	6	2%
	public health	104	10%	83	22%
	public health in combination with another non-medical study	58	6%	26	7%
	other non-medical study	56	5%	94	25%
General interest in working in the PHS (PHS interest)	yes	151	15%	88	24%
	rather yes than no	262	26%	136	37%
	rather no than yes	398	39%	115	31%
	no	176	17%	15	4%
	don’t know	34	3%	18	5%

**Table 2:** Responses to the question of whether or not the PHS is considered as an attractive employer as well as distribution of arguments in favor or against the PHS being attractive.

Wave	Field of Study	Perception on the PHS as Prospective Employer	Responded to the Question		Provided Valid Written Response		Provided ≥1 Reason Why the PHS Is Not Attractive		Provided ≥1 Reason Why the PHS Is Attractive	
			(n)	(%)	(n)	(%)	(n)	(%) *	(n)	(%) *
wave 1 and wave 2	Medicine	total	1045		948		683	72%	312	33%
		is attractive	300	29%	285	30%	20	7%	282	99%
		is **not** attractive	745	71%	663	70%	663	100%	30	5%
	PH&ONM	total	530		445		201	45%	278	62%
		is attractive	339	63%	280	63%	37	13%	274	98%
		is **not** attractive	191	36%	165	37%	164	99%	4	2%
wave 1	Medicine	total	865		785		579	74%	245	31%
		is attractive	232	27%	219	28%	13	6%	217	99%
		is **not** attractive	633	73%	566	72%	566	100%	28	5%
	PH&ONM	total	267		236		107	45%	142	60%
		is attractive	161	60%	141	60%	12	9%	139	99%
		is **not** attractive	106	40%	95	40%	95	100%	3	3%
wave 2	Medicine	total	180		163		104	64%	67	41%
		is attractive	68	38%	66	40%	7	11%	65	98%
		is not attractive	112	62%	97	60%	97	100%	2	2%
	PH&ONM	total	263		209		94	45%	136	65%
		is attractive	178	68%	139	67%	25	18%	135	97%
		is **not** attractive	85	32%	70	33%	69	99%	1	1%

* Relative proportion of valid written response, bold text to highlight the difference between the both categories.

**Table 3:** Main reasons provided for why working in the PHS was not considered attractive.

Level	Code	MS		PH&ONM	
		(n)	(%)	(n)	(%)
	at least one reason for not considering the PHS attractive, a statement related to a lack of knowledge about the PHS, or an unclear or ambiguous statement	683	100%	202	100%
	at least one reason for not considering the PHS attractive	651	95%	187	92%
	at least one statement related to a lack of knowledge about the PHS, or an unclear or ambiguous statement	174	26%	47	23%
1	reasons related to impact and effect	31	4%	11	5%
2	limited impact or not making a difference	31	4%	11	5%
1	reasons related to reputation and image	36	5%	15	7%
2	negative image and reputation of the PHS	36	5%	15	7%
1	reasons related to institution	192	28%	101	50%
2	bureaucratic institutions and work	172	25%	73	37%
2	outdated, not innovative, and in need of modernization	55	8%	58	28%
2	more attention to diversity and gender equity needed	0	0%	3	2%
1	reasons related to statements related to interest	326	47%	71	36%
2	participant is “just not interested”	136	19%	35	17%
2	no suggestion on how to improve the PHS	196	29%	36	17%
1	reasons related to job, employment, and employer	121	17%	53	27%
2	low accessibility and lacking acknowledgment for non-medical professionals	2	0%	22	10%
2	career opportunities are lacking or unattractive	34	5%	11	5%
2	working hours are unattractive	22	3%	13	6%
2	salary and remuneration are too low	87	12%	19	9%
1	reasons related to occupation	144	21%	50	25%
2	limited creative freedoms	34	5%	30	15%
2	not challenging, not for ambitious people	25	4%	8	4%
2	stress and workload are high	18	2%	3	2%
2	occupation is boring, monotonous, or generally not interesting	87	12%	15	7%
2	unattractive working conditions or working atmosphere	2	0%	1	1%
1	reasons related to responsiveness and localization	31	4%	21	11%
2	need to improve responsiveness to population	2	0%	9	5%
2	influence or restrictions by politics are too high	16	2%	6	3%
2	too local, need to strengthen international and global focus	13	1%	6	3%
1	reasons related to topics and activities	417	61%	33	16%
2	insufficient focus or impact on SDH systems or structures or on prevention and health promotion	22	3%	9	5%
2	not enough (clinical) medicine, too few patients	392	57%	9	5%
2	too much focus on medicine	0	0%	8	4%
2	not enough research	23	3%	9	5%
2	too much research	1	0%	1	1%
2	suggestion to focus on specific topics	7	1%	4	2%
1	statements related to a lack of knowledge about the PHS	167	24%	39	19%
2	lack of knowledge about the PHS	162	23%	36	17%
2	offer internships, be more present in curriculum	28	5%	5	3%
1	other unclear, ambiguous statements	8	1%	10	5%

This table is limited to reasons at level 1 and 2, with ≥5 individuals having made a statement in this regard among either medical or PH&ONM students (*Abbreviations: SDH: social determinants of health, MS: medical students, PH&ONM: public health and other non-medical students*).

**Table 4:** Main reasons provided for why working in the PHS was considered attractive.

Level	Code	MS		PH&ONM	
		(n)	(%)	(n)	(%)
	at least one reason for considering the PHS as attractive	312	100%	278	100%
	at least one reason for not considering the PHS as attractive (within this population)	45	14%	33	12%
1	reasons related to job, employment, and employer	185	59%	115	41%
2	attractive career opportunities	4	1%	5	2%
2	high degree of job security	40	13%	77	28%
2	good work-life balance and attractive working hours	158	51%	38	14%
2	attractive salary	8	3%	21	8%
1	reasons related to occupation	96	31%	101	36%
2	provides creative freedoms	11	4%	10	4%
2	challenging and allows to use one’s skills and abilities	1	0%	17	6%
2	job is relaxed, stress and overall workload is low	23	7%	12	4%
2	working conditions or working atmosphere are good	14	4%	3	1%
2	occupation is exciting, diverse, or generally interesting	52	17%	63	23%
2	interdisciplinary team structure	4	1%	5	2%
1	reasons related to topics and activities	99	32%	74	27%
2	action on prevention or on the level of population, systems, and structures (SDH)	67	21%	68	24%
2	alternative to clinical medicine	25	8%	2	1%
2	allows combining medicine and public health	6	2%	1	0%
1	reasons related to impact and effect	54	17%	63	23%
2	having an impact and making a difference	54	17%	63	23%
1	reasons related to responsiveness and localization	8	3%	12	4%
2	being active and having impact in the community	4	1%	12	4%

This table is limited to reasons at level 1 and 2, with ≥5 individuals having made a statement in this regard among either medical or PH&ONM students *(Abbreviations: SDH: social determinants of health, MS: medical students, PH&ONM: public health and other non-medical students)*.

## Data Availability

After project completion, the quantitative data of the OeGD-Studisurvey will be available on https://osf.io/uxftz (accessed on 1 July 2022). To prevent identification of individuals through the combination of the open-ended answers and socio-demographic data, the unredacted dataset will not be publicly available due to privacy reasons. The data presented in this study are available on request from the corresponding author.

## Supplementary Materials

The following supporting information can be downloaded at: https://www.mdpi.com/article/10.3390/ijerph191811733/s1, Table S1: S1_OeGD-Studisurvey_coding_frame.xlsx.

## Appendix A. List of Abbreviations

	**English**	**German**
AOEGW	Academy of Public Health Services	Akademie für Öffentliches Gesundheitswesen in Düsseldorf
bvmd	German Medical Students’ Association	Bundesvertretung der Medizinstudierenden in Deutschland e.V.
BVOEGD	Federal Association for Physicians in the PHS	Bundesverband für Ärztinnen und Ärzte im Öffentlichen Gesundheitsdienst
MS	medical students	Medizinstudierende
NOEG	German Network of Young Professionals in Public Health	Nachwuchsnetzwerk Oeffentliche Gesundheit
PH	public health	Public Health
PH&ONM	public health and other non-medical students	Public Health Studierende und Studierende anderer nicht-medizinischer Studiengänge
PHS	Public Health Service	Öffentlicher Gesundheitsdienst (in German: OEGD)
RKI	Robert Koch Institute	Robert Koch-Institut
SDH	social determinants of health	Soziale Determinanten von Gesundheit

## Appendix B. Additional and Extended Tables

**Table A1:** Reasons for not considering the PHS as an attractive employer – extended table.

Level	Code	MS		PH&ONM		Difference
		(n)	(%)	(n)	(%)	*p*-Value
	at least one reason for not considering the PHS attractive, a statement related to a lack of knowledge about the PHS, or an unclear or ambiguous statement	683	100%	202	100%	
	at least one reason for not considering the PHS attractive	651	95%	187	92%	
	at least one statement related to a lack of knowledge about the PHS, or an unclear or ambiguous statement	174	26%	47	23%	
	at least one reason for considering the PHS attractive (within this population)					
1	reasons related to impact and effect	31	4%	11	5%	
2	limited impact or not making a difference	31	4%	11	5%	
3	need to make the experience that PHS has an impact or work makes a difference	15	3%	1	1%	
3	to having impact or feeling to not make a difference	8	1%	2	1%	
3	suggestion to increasing impact, reach more people, or to expand sphere of influence	7	1%	2	1%	
3	low impact, reaching few people, narrow sphere of influence	3	0%	5	3%	***
1	reasons related to reputation and image	36	5%	15	7%	
2	negative image and reputation of the PHS	36	5%	15	7%	
3	need to improve image and reputation image, need to increase awareness about the PHS and its impact	23	3%	10	5%	
3	negative and reputation image, low awareness about the PHS	17	2%	4	2%	
1	reasons related to institution	192	28%	101	50%	****
2	bureaucratic institutions and work	172	25%	73	37%	****
3	too much bureaucracy, to bureaucratic (in general)	51	7%	30	15%	****
3	too much desk work, too much paperwork	37	5%	4	2%	*
3	reduce bureaucracy, be less bureaucratic (in general)	40	6%	10	5%	
3	inert, nondynamic, slow, inefficient	21	3%	11	5%	*
3	structures too rigid	18	2%	13	6%	*
3	no interest in working for a classical institution of public administration	15	3%	4	2%	
3	too much administrative work	11	1%	5	3%	
3	bound or restricted by rules and regulations	8	1%	4	2%	
3	bound or restricted by bureaucracy or bureaucratic structures	8	1%	5	3%	
3	inflexible and rigid hierarchies	5	0%	13	6%	****
3	too theoretical, too little practice	5	0%	8	4%	****
2	outdated, not innovative, and in need of modernization	55	8%	58	28%	****
3	outdated, not innovative, and in need of modernization (in general)	38	5%	45	23%	****
3	need for more digitalization	12	2%	8	4%	*
3	needs modern work and administrative structures	3	0%	8	4%	****
3	need for reform (in general)	2	0%	8	4%	****
2	more attention to diversity and gender equity needed	0	0%	3	2%	***
1	reasons related to statements related to interest	326	47%	71	36%	****
2	participant is “just not interested”	136	19%	35	17%	
3	participant is “just not interested”	136	19%	35	17%	
2	no suggestion on how to improve the PHS	196	29%	36	17%	****
3	no suggestion on how to improve the PHS	196	29%	36	17%	****
1	reasons related to job, employment, and employer	121	17%	53	27%	****
2	low accessibility and lacking acknowledgment for non-medical professionals	2	0%	22	10%	****
3	limited availability of jobs for individuals without a medical degree	0	0%	10	5%	****
3	limited accessibility of jobs for individuals without a medical degree	1	0%	8	4%	****
3	lacking acknowledgment of individuals without a medical degree	1	0%	10	5%	****
2	career opportunities are lacking or unattractive	34	5%	11	5%	
3	career opportunities are lacking or unattractive	28	5%	8	4%	
3	opportunities for continuous education are lacking or unattractive	6	1%	4	2%	
2	working hours are unattractive	22	3%	13	6%	*
3	needs to offer attractive working hours	18	2%	13	6%	***
2	salary and remuneration are too low	87	12%	19	9%	
3	salary is too low	85	12%	19	9%	
1	reasons related to occupation	144	21%	50	25%	
2	limited creative freedoms	34	5%	30	15%	****
3	need to increase creative freedoms	21	3%	13	6%	***
3	occupation does not provide sufficient creative freedoms	15	3%	19	9%	****
2	not challenging, not for ambitious people	25	4%	8	4%	
3	occupation is not challenging	12	2%	4	2%	
3	is not attractive for ambitious people	10	1%	3	2%	
2	stress and workload is high	18	2%	3	2%	
3	stress or workload is too high	17	2%	3	2%	
2	occupation is boring, monotonous, or generally not interesting	87	12%	15	7%	***
3	occupation is boring, monotonous, or generally not interesting	87	12%	15	7%	***
2	unattractive working conditions or working atmosphere	2	0%	1	1%	
1	reasons related to responsiveness and localization	31	4%	21	11%	****
2	need to improve responsiveness to population	2	0%	9	5%	****
3	need to be more in touch with the population	1	0%	7	4%	****
2	influence or restrictions by politics are too high	16	2%	6	3%	
3	too much bound or restricted by politics and politicians	8	1%	2	1%	
3	need to achieve more independence from politics	5	0%	5	3%	*
2	too local, need to strengthen international and global focus	13	1%	6	3%	
3	need to strengthen international and global focus	12	2%	1	1%	
1	reasons related to topics and activities	417	61%	33	16%	****
2	insufficient focus or impact on social determinants of health, affecting health on system or structural level or on prevention and health promotion	22	3%	9	5%	
3	need to focus more on influencing politics	8	1%	2	1%	
3	need to focus more on social determinants of health; on underlying systems or structures	6	1%	2	1%	
3	need to focus more on setting-based prevention and health promotion	8	1%	4	2%	
2	not enough (clinical) medicine, too few patients	392	57%	9	5%	****
3	not enough (clinical) medicine, too few patients	367	53%	4	2%	****
3	more contact to patients, more clinical practice	67	9%	1	1%	****
3	too much public health	12	2%	4	2%	
2	too much focus on medicine	0	0%	8	4%	****
3	too much focus on medicine	0	0%	8	4%	****
2	not enough research	23	3%	9	5%	
3	lack of research	11	1%	7	4%	
3	need to improve or provide opportunities for research	11	1%	2	1%	
2	too much research	1	0%	1	1%	
2	suggestion to focus on specific topics	7	1%	4	2%	
3	suggestion to focus on specific topics	7	1%	4	2%	
1	statements related to a lack of knowledge about the PHS	167	24%	39	19%	
2	lack of knowledge about the PHS	162	23%	36	17%	*
3	lack of knowledge about the PHS	162	23%	36	17%	*
2	offer internships, be more present in curriculum	28	5%	5	3%	
3	offer (more) internships	22	3%	4	2%	
3	be more present in curriculum	8	1%	1	1%	
1	other unclear, ambiguous statements	8	1%	10	5%	****

This table is limited to reasons at level 1 and 2 as well as those reasons at level 3, with ≥5 individuals having made a statement in this regard among either medical or PH&ONM students *(Abbreviations: SDH: social determinants of health, MS: medical students, PH&ONM: public health and other non-medical students)* *: *p*-value < 0.1; ***: *p*-value < 0.05; ****: *p*-value < 0.01.

**Table A2:** Reasons for considering the PHS as an attractive employer—extended table.

Level	Code	Wave 1					Wave 2					MS	PH&ONM
		MS		PH&ONM		Difference	MS		PH&ONM		Difference	Difference	
						MS vs. PH&ONM					MS vs. PH&ONM	Wave 1 vs. Wave 2	
		(n)	(%)	(n)	(%)	p-Value	(n)	(%)	(n)	(%)	p-Value	p-Value	p-Value
	at least one reason for not considering the PHS attractive, a statement related to a lack of knowledge about the PHS, or an unclear or ambiguous statement	579	100%	107	100%		104	100%	95	100%			
	at least one reason for not considering the PHS attractive	554	95%	98	91%	*	97	93%	89	93%			
	at least one statement related to a lack of knowledge about the PHS, or an unclear or ambiguous statement	152	26%	29	27%		22	21%	18	18%			
	at least one reason for considering the PHS attractive (within this population)												
1	reasons related to impact and effect	28	4%	6	5%		3	2%	5	5%			
2	limited impact or not making a difference	28	4%	6	5%		3	2%	5	5%			
3	need to make the experience that PHS has an impact or work makes a difference	12	2%	0	0%		3	2%	1	1%			
3	to having impact or feeling to not make a difference	7	1%	2	1%		1	1%	0	0%			
3	suggestion to increasing impact, reach more people, or to expand sphere of influence	7	1%	2	1%		0	0%	0	0%			
1	reasons related to reputation and image	29	5%	9	8%		7	6%	6	6%			
2	negative image and reputation of the PHS	29	5%	9	8%		7	6%	6	6%			
3	need to improve image and reputation image, need to increase awareness about the PHS and its impact	19	3%	4	3%		4	3%	6	6%			
3	negative and reputation image, low awareness about the PHS	13	2%	4	3%		4	3%	0	0%			
1	reasons related to institution	157	27%	52	48%	****	35	33%	49	51%	***		
2	bureaucratic institutions and work	142	24%	40	37%	***	30	28%	33	34%			
3	too much bureaucracy, to bureaucratic (in general)	44	7%	17	15%	***	7	6%	13	13%			
3	too much desk work, too much paperwork	33	5%	0	0%	***	4	3%	4	4%			***
3	reduce bureaucracy, be less bureaucratic (in general)	32	5%	4	3%		8	7%	6	6%			
3	inert, nondynamic, slow, inefficient	17	2%	4	3%		4	3%	7	7%			
3	structures too rigid	14	2%	5	4%		4	3%	8	8%			
3	no interest in working for a classical institution of public administration	11	1%	2	1%		4	3%	2	2%			
3	too much administrative work	9	1%	2	1%		2	1%	3	3%			
3	bound or restricted by rules and regulations	7	1%	3	2%		1	1%	1	1%			
3	bound or restricted by bureaucracy or bureaucratic structures	6	1%	3	2%		2	1%	2	2%			
3	inflexible and rigid hierarchies	5	0%	11	10%	****	0	0%	2	2%			***
3	too theoretical, too little practice	5	0%	3	2%		0	0%	5	5%	***		
2	outdated, not innovative, and in need of modernization	41	7%	27	25%	****	14	13%	31	32%	***	***	
3	outdated, not innovative, and in need of modernization (in general)	27	4%	20	18%	****	11	10%	25	26%	***	***	
3	need for more digitalization	7	1%	3	2%		5	4%	5	5%		***	
3	needs modern work and administrative structures	3	0%	2	1%		0	0%	6	6%	***		
3	need for reform (in general)	2	0%	2	1%		0	0%	6	6%	***		
2	more attention to diversity and gender equity needed	0	0%	0	0%		0	0%	3	3%			
1	reasons related to statements related to interest	287	49%	42	39%	*	39	37%	29	30%		***	
2	participant is “just not interested”	121	20%	22	20%		15	14%	13	13%			
3	participant is “just not interested”	121	20%	22	20%		15	14%	13	13%			
2	no suggestion on how to improve the PHS	170	29%	20	18%	***	26	25%	16	16%			
3	no suggestion on how to improve the PHS	170	29%	20	18%	***	26	25%	16	16%			
1	reasons related to job, employment, and employer	98	16%	24	22%		23	22%	29	30%			
2	low accessibility and lacking acknowledgment for non-medical professionals	1	0%	7	6%	****	1	1%	15	15%	****		***
3	limited availability of jobs for individuals without a medical degree	0	0%	4	3%	****	0	0%	6	6%	***		
3	limited accessibility of jobs for individuals without a medical degree	0	0%	3	2%	***	1	1%	5	5%			
3	lacking acknowledgment of individuals without a medical degree	1	0%	3	2%	***	0	0%	7	7%	***		
2	career opportunities are lacking or unattractive	25	4%	6	5%		9	8%	5	5%		*	
3	career opportunities are lacking or unattractive	21	3%	5	4%		7	6%	3	3%			
2	working hours are unattractive	19	3%	6	5%		3	2%	7	7%			
3	needs to offer attractive working hours	15	2%	6	5%		3	2%	7	7%			
2	salary and remuneration is too low	71	12%	9	8%		16	15%	10	10%			
3	salary is too low	70	12%	9	8%		15	14%	10	10%			
1	reasons related to occupation	117	20%	29	27%		27	26%	21	22%			
2	limited creative freedoms	24	4%	19	17%	****	10	9%	11	11%		***	
3	need to increase creative freedoms	14	2%	8	7%	***	7	6%	5	5%		***	
3	occupation does not provide sufficient creative freedoms	12	2%	12	11%	****	3	2%	7	7%			
2	not challenging, not for ambitious people	19	3%	5	4%		6	5%	3	3%			
3	occupation is not challenging	9	1%	3	2%		3	2%	1	1%			
3	is not attractive for ambitious people	8	1%	1	0%		2	1%	2	2%			
2	stress and workload are high	14	2%	0	0%		4	3%	3	3%			
3	stress or workload is too high	13	2%	0	0%		4	3%	3	3%			
2	occupation is boring, monotonous, or generally not interesting	73	12%	10	9%		14	13%	5	5%	*		
3	occupation is boring, monotonous, or generally not interesting	73	12%	10	9%		14	13%	5	5%	*		
2	unattractive working conditions or working atmosphere	1	0%	0	0%		1	1%	1	1%			
1	reasons related to responsiveness and localization	23	4%	11	10%	***	8	7%	10	10%			
2	need to improve responsiveness to population	1	0%	2	1%	*	1	1%	7	7%	***		*
3	need to be more in touch with the population	0	0%	1	0%		1	1%	6	6%	*		*
2	influence or restrictions by politics are too high	10	1%	3	2%		6	5%	3	3%		***	
3	too much bound or restricted by politics and politicians	5	0%	1	0%		3	2%	1	1%			
3	need to achieve more independence from politics	0	0%	2	1%	***	5	4%	3	3%		****	
2	too local, need to strengthen international and global focus	12	2%	6	5%	***	1	1%	0	0%			***
3	need to strengthen international and global focus	12	2%	1	0%		0	0%	0	0%			
1	reasons related to topics and activities	360	62%	22	20%	****	57	54%	11	11%	****		*
2	insufficient focus or impact on social determinants of health, affecting health on system or structural level or on prevention and health promotion	21	3%	5	4%		1	1%	4	4%			
3	need to focus more on influencing politics	8	1%	1	0%		0	0%	1	1%			
3	need to focus more on social determinants of health; on underlying systems or structures	6	1%	1	0%		0	0%	1	1%			
3	need to focus more on setting-based prevention and health promotion	8	1%	4	3%		0	0%	0	0%			
2	not enough (clinical) medicine, too few patients	339	58%	7	6%	****	53	51%	2	2%	****		
3	not enough (clinical) medicine, too few patients	318	54%	3	2%	****	49	47%	1	1%	****		
3	more contact to patients, more clinical practice	61	10%	0	0%	****	6	5%	1	1%			
3	too much public health	11	1%	4	3%		1	1%	0	0%			
2	too much focus on medicine	0	0%	5	4%	****	0	0%	3	3%			
3	too much focus on medicine	0	0%	5	4%	****	0	0%	3	3%			
2	not enough research	16	2%	6	5%		7	6%	3	3%		*	
3	lack of research	8	1%	5	4%	***	3	2%	2	2%			
2	need to improve or provide opportunities for research	8	1%	1	0%		3	2%	1	1%			
2	too much research	1	0%	1	0%		0	0%	0	0%			
2	suggestion to focus on specific topics	6	1%	1	0%		1	1%	3	3%			
3	suggestion to focus on specific topics	6	1%	1	0%		1	1%	3	3%			
1	statements related to a lack of knowledge about the PHS	146	25%	24	22%		21	20%	15	15%			
2	lack of knowledge about the PHS	142	24%	22	20%		20	19%	14	14%			
3	lack of knowledge about the PHS	142	24%	22	20%		20	19%	14	14%			
2	offer internships, be more present in curriculum	25	4%	3	2%		3	2%	2	2%			
3	offer (more) internships	19	3%	2	1%		3	2%	2	2%			
3	be more present in curriculum	8	1%	1	0%		0	0%	0	0%			
1	other unclear, ambiguous statements	7	1%	7	6%	***	1	1%	3	3%			

This table is limited to reasons at level 1 and 2 as well as those reasons at level 3, with ≥5 individuals having made a statement in this regard among either medical or PH&ONM students; *(Abbreviations: SDH: social determinants of health, MS: medical students, PH&ONM: public health and other non-medical students)* *: *p*-value < 0.1; ***: *p*-value < 0.05 ; ****: *p*-value < 0.001.

**Table A3:** Reasons for not considering the PHS as an attractive employer—disaggregated by wave 1 and wave 2.

Level	Codes	MS		PH&ONM		Difference
		(n)	(%)	(n)	(%)	*p*-Value
	at least one reason for considering the PHS as attractive	312	100%	278	100%	
	at least one reason for not considering the PHS as attractive (within this population)	45	14%	33	12%	
	at least one statement related to a lack of knowledge about the PHS	9	3%	6	2%	
1	reasons related to job, employment, and employer	185	59%	115	41%	****
2	attractive career opportunities	4	1%	5	2%	
2	high degree of job security	40	13%	77	28%	****
3	high degree of job security (in general)	29	9%	69	25%	****
3	working for public service is attractive	1	0%	6	2%	*
3	provides chance to become a public servant (Ger: Beamter)	11	4%	4	1%	
2	good work-life balance and attractive working hours	158	51%	38	14%	****
3	working hours are flexible	6	2%	6	2%	
3	working hours are predictable	68	22%	9	3%	****
3	provides attractive work-life balance	32	10%	15	5%	***
3	length of working days and hours are attractive	20	6%	1	0%	****
3	working hours or employer is family friendly	47	15%	11	4%	****
3	no shift-work, no need to work at night or on weekends	24	8%	0	0%	****
2	attractive salary	8	3%	21	8%	****
3	attractive salary	8	3%	21	8%	****
2	availability and accessibility of jobs	1	0%	4	1%	
2	sensitive and open to diversity and diverse needs	2	1%	0	0%	
1	reasons related to occupation	96	31%	101	36%	*
2	provides creative freedoms	11	4%	10	4%	
3	provides creative freedoms	6	2%	8	3%	
2	challenging and allows to use one’s skills and abilities	1	0%	17	6%	****
3	occupations are challenging	0	0%	8	3%	****
3	allows to use one’s skills and abilities	1	0%	9	3%	****
2	job is relaxed, stress and overall workload is low	23	7%	12	4%	
3	stress and overall workload are low	4	1%	6	2%	
3	job is relaxed	17	5%	3	1%	***
2	working conditions or working atmosphere are good	14	4%	3	1%	***
3	working conditions are good	8	3%	3	1%	
3	working atmosphere are good	6	2%	0	0%	***
2	occupation is exciting, diverse, or generally interesting	52	17%	63	23%	*
3	occupation is exciting, diverse, or generally interesting	44	14%	54	19%	*
3	interesting due to interest in specific topic	8	3%	10	4%	
2	flat hierarchies	3	1%	0	0%	
3	interdisciplinary team structure	4	1%	5	2%	
2	interdisciplinary team structure	4	1%	5	2%	
1	reasons related to topics and activities	99	32%	74	27%	
2	action on prevention or on the level of population, systems, and structures (SDH)	67	21%	68	24%	
3	action on a population level	21	7%	33	12%	***
3	attractive, due to being interested in public health	9	3%	8	3%	
3	has a broader, more holistic perspective on health	6	2%	4	1%	
3	action on SDHs or on the system or structural level	19	6%	18	6%	
3	prevention focused	19	6%	7	3%	***
3	focused on setting-based prevention and health promotion	5	2%	11	4%	*
2	alternative to clinical medicine	25	8%	2	1%	****
3	good alternative to clinic	17	5%	1	0%	****
3	backup option to clinic	9	3%	1	0%	***
2	allows combining medicine and public health	6	2%	1	0%	
3	allows combining medicine and public health	6	2%	1	0%	
2	ability to do research	4	1%	3	1%	
1	reasons related to impact and effect	54	17%	63	23%	
2	having an impact and making a difference	54	17%	63	23%	
3	has an impact or (experience of) making a difference	12	4%	19	7%	
3	high impact, reaching many people, broad sphere of influence	25	8%	12	4%	*
3	PHS and its work is important	11	4%	25	9%	****
3	having a positive impact on vulnerable populations	4	1%	7	3%	
1	reasons related to responsiveness and localization	8	3%	12	4%	
2	has a global perspective, focus, or impact	4	1%	0	0%	
2	being active and having impact in the community	4	1%	12	4%	***
3	acting in the community or on the local level	3	1%	9	3%	*
1	reasons related to reputation and image	1	0%	1	0%	
2	positive image and good reputation	1	0%	1	0%	
1	reasons related to institution	3	1%	4	1%	
2	willingness to change	3	1%	4	1%	

This table is limited to reasons at level 1 and 2 as well as those reasons at level 3, with ≥5 individuals having made a statement in this regard among either medical or PH&ONM students; *(Abbreviations: SDH: social determinants of health, MS: medical students, PH&ONM: public health and other non-medical students)* *: *p-*value < 0.1; ***: *p*-value < 0.05; ****: *p*-value < 0.01.

**Table A4:** Reasons for considering the PHS as an attractive employer—disaggregated by wave 1 and wave 2.

Level	Code	Wave 1					Wave 2					MS	PH&ONM
		MS		PH&ONM		Difference	MS		PH&ONM		Difference	Difference	
						MS vs. PH&ONM					MS vs. PH&ONM	Wave 1 vs. Wave 2	
		(n)	(%)	(n)	(%)	*p*-Value	(n)	(%)	(n)	(%)	*p-*Value	*p-*Value	*p-*Value
	at least one reason for considering the PHS as attractive	245	100%	142	100%		67	100%	136	100%			
	at least one reason for not considering the PHS as attractive (within this population)	39	16%	12	9%	***	6	9%	21	15%			*
	at least one statement related to a lack of knowledge about the PHS	5	2%	2	1%		4	6%	4	3%			
1	reasons related to impact and effect	45	18%	34	24%		9	13%	29	21%			
2	having an impact and making a difference	45	18%	34	24%		9	13%	29	21%			
3	has an impact or (experience of) making a difference	11	5%	9	6%		1	2%	10	7%			
3	high impact, reaching many people, broad sphere of influence	22	9%	7	5%		3	5%	5	4%			
3	PHS and its work is important	7	3%	14	10%	***	4	6%	11	8%			
1	reasons related to reputation and image	0	0%	0	0%		1	2%	1	1%			
2	positive image and good reputation	0	0%	0	0%		1	2%	1	1%			
1	reasons related to institution	3	1%	4	3%		0	0%	0	0%			
2	willingness to change	3	1%	4	3%		0	0%	0	0%			
1	reasons related to job, employment, and employer	139	57%	43	30%	****	46	69%	72	53%	***	*	****
2	attractive career opportunities	2	1%	1	1%		2	3%	4	3%			
2	high degree of job security	30	12%	30	21%	***	10	15%	47	35%	***		***
3	high degree of job security (in general)	20	8%	28	20%	****	9	13%	41	30%	***		*
3	provides chance to become a public servant (Ger: Beamter)	9	4%	0	0%	***	2	3%	4	3%			*
2	good work-life balance and attractive working hours	118	48%	14	10%	****	40	60%	24	18%	****	*	*
3	working hours are predictable	60	25%	6	4%	****	8	12%	3	2%	***	***	
3	provides attractive work-life balance	16	7%	2	1%	***	16	24%	13	10%	***	****	***
3	length of working days and hours are attractive	12	5%	0	0%	***	8	12%	1	1%	****	***	
3	working hours or employer is family friendly	36	15%	6	4%	****	11	16%	5	4%	***		
3	no shift-work, no need to work at night or on weekends	16	7%	0	0%	****	8	12%	0	0%	****		
2	attractive salary	5	2%	8	6%	*	3	5%	13	10%			
3	attractive salary	5	2%	8	6%	*	3	5%	13	10%			
2	availability and accessibility of jobs	1	0%	0	0%		0	0%	4	3%			*
2	sensitive and open to diversity and diverse needs	1	0%	0	0%		1	2%	0	0%			
1	reasons related to occupation	73	30%	52	37%		23	34%	49	36%			
2	provides creative freedoms	9	4%	4	3%		2	3%	6	4%			
3	provides creative freedoms	4	2%	3	2%		2	3%	5	4%			
2	challenging and allows to use one’s skills and abilities	1	0%	11	8%	****	0	0%	6	4%			
3	occupations are challenging	0	0%	5	4%	***	0	0%	3	2%			
3	allows to use one’s skills and abilities	1	0%	6	4%	***	0	0%	3	2%			
2	job is relaxed, stress and overall workload is low	16	7%	5	4%		7	10%	7	5%			
3	job is relaxed	13	5%	1	1%	***	4	6%	2	2%	*		
2	working conditions or working atmosphere are good	11	5%	3	2%		3	5%	0	0%	***		
3	working conditions are good	8	3%	3	2%		0	0%	0	0%			
2	occupation is exciting, diverse, or generally interesting	40	16%	32	23%		12	18%	31	23%			
3	occupation is exciting, diverse, or generally interesting	32	13%	29	20%	*	12	18%	25	18%			
3	interesting due to interest in specific topic	8	3%	3	2%		0	0%	7	5%	*		
2	flat hierarchies	2	1%	0	0%		1	2%	0	0%			
2	interdisciplinary team structure	3	1%	3	2%		1	2%	2	2%			
1	reasons related to responsiveness and localization	8	3%	9	6%		0	0%	3	2%			
2	has a global perspective, focus, or impact	4	2%	0	0%		0	0%	0	0%			
2	being active and having impact in the community	4	2%	9	6%	***	0	0%	3	2%			
3	acting in the community or on the local level	3	1%	6	4%	*	0	0%	3	2%			
1	reasons related to topics and activities	76	31%	41	29%		23	34%	33	24%			
2	action on prevention or on the level of population, systems, and structures (SDH)	49	20%	39	28%		18	27%	29	21%			
3	action on a population level	16	7%	15	11%		5	8%	18	13%			
3	attractive, due to being interested in public health	8	3%	7	5%		1	2%	1	1%			*
3	has a broader, more holistic perspective on health	6	2%	2	1%		0	0%	2	2%			
3	action on SDHs or on the system or structural level	14	6%	11	8%		5	8%	7	5%			
3	prevention focused	11	5%	4	3%		8	12%	3	2%	***	***	
3	focused on setting-based prevention and health promotion	3	1%	8	6%	***	2	3%	3	2%			
2	alternative to clinical medicine	22	9%	1	1%	****	3	5%	1	1%			
3	good alternative to clinic	14	6%	0	0%	***	3	5%	1	1%			
3	backup option to clinic	9	4%	1	1%	*	0	0%	0	0%			
2	allows combining medicine and public health	5	2%	0	0%		1	2%	1	1%			
3	allows combining medicine and public health	5	2%	0	0%		1	2%	1	1%			
2	ability to do research	1	0%	1	1%		3	5%	2	2%		***	

This table is limited to reasons at level 1 and 2 as well as those reasons at level 3, with ≥5 individuals having made a statement in this regard among either medical or PH&ONM students; (*Abbreviations: SDH: social determinants of health, MS: medical students, PH&ONM: public health and other non-medical students*) *: *p*-value < 0.1; ***: *p*-value < 0.05; ****: *p*-value < 0.001.

**Table A5:** Attractiveness of the PHS by previous experience in the PHS: overview.

		Total	At Least One Reason for Considering the PHS Attractive			At Least One Reason for Not Considering the PHS Attractive		
		[N]	(n)	(%)	*p-*Value ^1^	(n)	(%)	*p-*Value ^1^
Medical	previous experience in the PHS	48	23	48%	-	28	58%	-
students	no previous practical experience in the PHS	115	44	38%	-	76	66%	-
PH& ONM	previous experience in the PHS	105	73	70%	-	42	40%	-
students	no previous practical experience in the PHS	104	62	60%	-	53	51%	-

^1^ Pearson chi-square statistic to assess the difference in proportions of individuals providing at least one reason for considering the PHS (not) attractive by self-reported previous experience in the PHS.

**Table A6:** (**a**,**b**) Reasons for not considering PHS attractive by previous experience within the PHS.

(a)																
Level	Code	MS and PH&ONM					MS					PH&ONM				
		No Exp.		Exp.		Difference	No Exp.		Exp.		Difference	No exp.		Exp.		Difference
		(n)	(%)	(n)	(%)	*p-*Value	(n)	(%)	(n)	(%)	*p-*Value	(n)	(%)	(n)	(%)	*p-*Value
	at least one reason for not considering the PHS attractive, a statement related to a lack of knowledge about the PHS, or an unclear or ambiguous statement	129	100%	70	100%		76	100%	28	100%		53	100%	42	100%	
	at least one reason for not considering the PHS attractive	121	94%	65	93%		71	93%	26	93%		50	94%	39	93%	
	at least one statement related to a lack of knowledge about the PHS, or an unclear or ambiguous statement	28	22%	12	17%		17	22%	5	18%		11	21%	7	17%	
	at least one reason for considering the PHS attractive (within this population)	18	14%	13	19%		5	7%	3	11%		13	25%	10	24%	
1	reasons related to institution	55	43%	29	41%		25	33%	10	36%		30	57%	19	45%	
2	bureaucratic institutions and work	41	32%	22	31%		20	26%	10	36%		21	40%	12	29%	
2	outdated, not innovative, and in need of modernization	28	22%	17	24%		12	16%	2	7%		16	30%	15	36%	
2	more attention to diversity and gender equity needed	3	2%	0	0%		0	0%	0	0%		3	6%	0	0%	
1	reasons related to topics and activities	50	39%	18	26%	*	44	58%	13	46%		6	11%	5	12%	
2	insufficient focus on social determinants of health, affecting health on system or structural level or on prevention and health promotion	3	2%	2	3%		1	1%	0	0%		2	4%	2	5%	
2	not enough (clinical) medicine, too few patients	43	33%	12	17%	***	42	55%	11	39%		1	2%	1	2%	
2	too much focus on medicine	2	2%	1	1%		0	0%	0	0%		2	4%	1	2%	
2	not enough research	4	3%	6	9%	*	3	4%	4	14%	*	1	2%	2	5%	
2	suggestion to focus on specific topics	3	2%	1	1%		1	1%	0	0%		2	4%	1	2%	
1	reasons related to statements related to interest	46	36%	22	31%		27	36%	12	43%		19	36%	10	24%	
2	participant is “just not interested”	20	16%	8	11%		11	14%	4	14%		9	17%	4	10%	
2	no suggestion on how to improve the PHS	26	20%	16	23%		16	21%	10	36%		10	19%	6	14%	
1	reasons related to job, employment, and employer	33	26%	19	27%		18	24%	5	18%		15	28%	14	33%	
2	low access. and lacking acknowledgment for non-medical professionals	10	8%	6	9%		0	0%	1	4%		10	19%	5	12%	
2	career opportunities are lacking or unattractive	9	7%	5	7%		8	11%	1	4%		1	2%	4	10%	
2	working hours are unattractive	5	4%	5	7%		2	3%	1	4%		3	6%	4	10%	
2	salary and remuneration are too low	18	14%	8	11%		13	17%	3	11%		5	9%	5	12%	
1	statements related to lack of knowledge about the PHS	27	21%	9	13%		17	22%	4	14%		10	19%	5	12%	
2	lack of knowledge about the PHS	26	20%	8	11%		17	22%	3	11%		9	17%	5	12%	
2	offer internships, be more present in curriculum	3	2%	2	3%		1	1%	2	7%		2	4%	0	0%	
1	reasons related to occupation	31	24%	17	24%		20	26%	7	25%		11	21%	10	24%	
2	limited creative freedoms	13	10%	8	11%		7	9%	3	11%		6	11%	5	12%	
2	not challenging, not for ambitious people	7	5%	2	3%		5	7%	1	4%		2	4%	1	2%	
2	stress and workload are high	5	4%	2	3%		4	5%	0	0%		1	2%	2	5%	
2	occupation is boring, monotonous, or generally not interesting	14	11%	5	7%		11	14%	3	11%		3	6%	2	5%	
2	unattractive working conditions or working atmosphere	2	2%	0	0%		1	1%	0	0%		1	2%	0	0%	
1	reasons related to responsiveness and localization	12	9%	6	9%		8	11%	0	0%	*	4	8%	6	14%	
2	need to improve responsiveness to population	3	2%	5	7%		1	1%	0	0%		2	4%	5	12%	
2	influence or restrictions by politics are too high	8	6%	1	1%		6	8%	0	0%		2	4%	1	2%	
2	too local, need to strengthen international and global focus	1	1%	0	0%		1	1%	0	0%		0	0%	0	0%	
1	reasons related to impact and effect	6	5%	2	3%		3	4%	0	0%		3	6%	2	5%	
1	reasons related to reputation and image	9	7%	4	6%		7	9%	0	0%		2	4%	4	10%	
1	other unclear, ambiguous statements	1	1%	3	4%		0	0%	1	4%		1	2%	2	5%	
**(b)**																
**Level**	**Code**	**MS and PH&ONM**					**MS**					**PH&ONM**				
		**No Exp.**		**Exp.**		**Difference**	**No Exp.**		**Exp.**		**Difference**	**No Exp.**		**Exp.**		**Difference**
		**(n)**	**(%)**	**(n)**	**(%)**	***p-*Value**	**(n)**	**(%)**	**(n)**	**(%)**	***p-*Value**	**(n)**	**(%)**	**(n)**	**(%)**	***p-*Value**
0	at least one reason for considering the PHS attractive	106	100%	96	100%		44	100%	23	100%		62	100%	73	100%	
	at least one reason for not considering the PHS attractive (within this population)	14	13%	13	14%		3	7%	3	18%		11	18%	10	14%	
	at least one statement related to a lack of knowledge about the PHS or an unclear or ambiguous statement	7	7%	1	1%	*	3	7%	1	6%		4	6%	0	0%	***
1	reasons related to job, employment, and employer	57	54%	61	64%		29	66%	17	45%		28	45%	44	60%	*
2	attractive career opportunities	3	3%	3	3%		0	0%	2	5%		3	5%	1	1%	
2	reasons related to high degree of job security	21	20%	36	38%	****	5	11%	5	26%		16	26%	31	42%	***
2	good work-life balance and attractive working hours	35	33%	29	30%		25	57%	15	16%		10	16%	14	19%	
2	attractive salary	7	7%	9	9%		1	2%	2	10%		6	10%	7	10%	
2	availability and accessibility of jobs	1	1%	3	3%		0	0%	0	2%		1	2%	3	4%	
2	sensitive and open to diversity and diverse needs	0	0%	1	1%		0	0%	1	0%		0	0%	0	0%	
1	reasons related to occupation	37	35%	35	36%		13	30%	10	39%		24	39%	25	34%	
2	provides creative freedoms	4	4%	4	4%		1	2%	1	5%		3	5%	3	4%	
2	challenging and allows to use one’s skills and abilities	4	4%	2	2%		0	0%	0	6%		4	6%	2	3%	
2	job is relaxed, stress and overall workload is low	2	2%	12	13%	****	2	5%	5	0%	***	0	0%	7	10%	***
2	working conditions or working atmosphere are good	1	1%	2	2%		1	2%	2	0%		0	0%	0	0%	
2	occupation is exciting, diverse, or generally interesting	25	24%	18	19%		8	18%	4	27%		17	27%	14	19%	
2	flat hierarchies	1	1%	0	0%		1	2%	0	0%		0	0%	0	0%	
1	reasons related to topics and activities	38	36%	18	19%	****	19	43%	4	31%	*	19	31%	14	19%	
2	action on prevention or on the level of population, systems, and structures (SDH)	34	32%	13	14%	****	16	36%	2	29%	***	18	29%	11	15%	*
2	alternative to clinical medicine	2	2%	2	2%		2	5%	1	0%		0	0%	1	1%	
2	allows combining medicine and public health	1	1%	1	1%		1	2%	0	0%		0	0%	1	1%	
2	ability to do research	3	3%	2	2%		2	5%	1	2%		1	2%	1	1%	
1	reasons related to impact and effect	24	23%	14	15%		6	14%	3	29%		18	29%	11	15%	*
2	having an impact and making a difference	24	23%	14	15%		6	14%	3	29%		18	29%	11	15%	*
1	reasons related to reputation and image	1	1%	1	1%		0	0%	1	2%		1	2%	0	0%	
2	positive image and good reputation	1	1%	1	1%		0	0%	1	2%		1	2%	0	0%	
1	responsiveness and localization	2	2%	1	1%		0	0%	0	3%		2	3%	1	1%	
2	has a global perspective, focus, or impact	0	0%	0	0%		0	0%	0	0%		0	0%	0	0%	
2	being active and having impact in the community	2	2%	1	1%		0	0%	0	3%		2	3%	1	1%	

This table is limited to reasons at level 1 and 2, with ≥ 1 individual having made a statement in this regard among either medical or PH&ONM students; (*Abbreviations: SDH: social determinants of health, MS: medical students, PH&ONM: public health and other non-medical students*) *: *p*-value < 0.1; ***: *p*-value < 0.05; ****: *p*-value < 0.01.

## Appendix C. Original Quotes in German

**Table A7:** Quotes stating why or why not working in the PHS was considered attractive.

**Quotes stating why working in the PHS was considered not attractive**	
**Insufficient knowledge**	
*“Unklar ist, was der ÖGD bietet, welche Aufgabenbereiche genau dazu gehören, die Tranzsparenz fehlt und unklar ist, inwiefern man nach einer Festanstellung beim ÖGD eine aktive Laufbahn am Patienten vor sich haben kann....* *”*	*(W1. ID467)*
*Patienten-fern (kaum direkter, länger anhaltender, therapeutischer Pat.-Kontakt); Bürokratie (glaube ich-hatte bisher eigentlich keinen Einblick). […] Bisher kaum Einblick, deshalb: Überhaupt erstmal präsent werden, im Studium thematisiert werden, sich interessant darstellen*	*(W1. ID496)*
*“Bisher kaum Einblick, deshalb: Überhaupt erstmal präsent werden, im Studium thematisiert werden, sich interessant darstellen* *”*	*(W1. ID710)*
*“Ich denke, es sollte mehr über die praktischen Aufgaben des ÖGD berichtet werden. Unter “Hygienemanagement” und “Trinkwasserüberwachung” kann ich mir keine konkreten Tätigkeiten vorstellen-und die Oberbegriffe klingen für mich als spätere Tätigkeit eher langweilig* *”*	*(W1. ID856)*
**Categorical lack of interest in working in the PHS or no suggestion on how to increase the attractiveness**	
*“es nicht unbedingt meinen interessen entspricht* *”*	*(W1. ID910)*
*“ich andere konkrete beruflichen Ziele anstrebe und diese mit einer Tätigkeit im ÖGD nicht zusammenpassen* *”*	*(W1. ID878)*
*“... ich in Richtung Forschnung und Entwicklung von Medikamenten gehen möchte. Meine Ziele für die Berufswahl müssten sich ändern. :)”*	*(W1. ID1016)*
*Not enough clinical medicine and contacts with patients*	
*“Weil ich* *in erster Linie als praktizierende Ärztin mit erkrankten Patienten in der Klinik arbeiten möchte. [...] Konkrete Patientenbehandlung müsste stets meine Hauptaufgabe sein”*	*(W1. ID447)*
*“Ich glaube, dass die Arbeit nicht genügend medizinische Praxis beinhaltet [...] Nichts, wirklich. Das Bild, dass ich vom ÖGD habe, beinhaltet wenig klinisch Praxis, und da ich behandelnder Arzt werden möchte, ist dieses Berufsfeld eher nichts für mich* *”*	*(W1. ID774)*
**Too bureaucratic work and institutions**	
*“man im ÖGD nur Verwaltungsaufgaben macht und die Leute, die im ÖGD arbeiten sehr unzufrieden sind* *”*	*(W1. ID549)*
*“zu viel Büokratie und starke Hierarchie wenig (Möglichkeit eigene Ideen einzubringen), Effekte/Erfolge werden nicht direkt erkennbar* *”*	*(W1. ID405)*
*“Es meiner Meinung nach zu viele Vorschriften und Gesetze gibt in Kombination mit Bürokratie die man lösen muss. Außerdem müsste ich dann auch viele Verwaltungsaufgaben übernehmen, was für mich eher uninteressant ist* *”*	*(W1. ID918)*
*“die Bürokratie zu ineffizient und zu langsam arbeitet, als dass man wirklich etwas verändern/gestalten könnte* *”*	*(W1. ID964)*
*“zu wenig echte Erfolge, durch hohe Bürokratie etc; Gefühl nichts zu erreichen durch zahlreiche Auflagen“*	*(W1. ID390)*
*“ich nicht im Büro sitzen möchte, Gutachten schreiben und nur über Zuständigkeiten diskutieren möchte (dafür ist der andere zuständig, Hauptsache wenig Arbeit)* *”*	*(W1. ID645)*
**Outdated, not innovative, and in need for modernization**	
*“Er müsste sich innovativ, modern, flexibel und vor allem unbürokratisch geben* *”*	*(W1. ID865)*
*“Stärkung der Digitalisierung und effiziente Umsetzung von neuen Ideen* *”*	*(W2. ID197)*
*“Deutschland ist unattraktiv, weil extrem hinterher und strukturell katastrophal in den Bereichen Public und Global Health, Innovation im Gesundheitsbereich/-wesen [und] transdisziplinäre Forschung* *”*	*(W1. ID968)*
**Occupation lacking creative freedoms**	
*“ich unabhängiger von öffentlichen Bestimmungen und Reglementierungen von Arbeitgebern meine eigenen Ideen und Vorstellungen in der Arbeit einbringen möchte* *”*	*(W1. ID624)*
*“zu viel Bürokratie und starke Hierarchie* *”*	*(W1. ID405)*
**PHS not providing job opportunities for and/or sufficient acknowledgement of individuals without a medical degree**	
*“Der ÖGD braucht mehr Platz auch für nicht ärztliches Personal* *”*	*(W2. ID141)*
*“es keine Arbeitsplätze für nicht-Mediziner:innen gibt* *”*	*(W2. ID353)*
**Salary and remuneration are too low**	
*“man nichts erreichen kann, was politisch nicht opportun ist und die Bezahlung die eigene Qualifikation nicht im Ansatz widerspiegelt (für ein Gehalt was geringer oder maximal dem eines Gymnasiallehrers entspricht jemanden mit Facharzttitel gewinnen zu wollen ist einfach lachhaft)* *”*	*(W1. ID918)*
*“Die Vergütung und Wertschätzung der Ärztinnen und Ärzte im ÖGD vollkommen unterdurchschnittlich sind. […]. Definitiv müsste es einen Ärztetarif innerhalb des ÖGD geben, der die Differenzen in der Vergütung gegenüber Klinikärzten aufhebt* *”*	*(W1. ID956)*
**Occupation being boring, monotonous, or generally not being interesting**	
*“Mir müsste bewiesen werden, dass der ÖGD eine bunte Palette an Arbeitsfeldern hat, innovativ und aktiv Forschung vorantreibt und tatsächlich die Politik beeinflusst. Man könnte auch Sagen, der ÖGD zeigen sollte dass er nicht nur aus zähen, trockenen und ineffizienten Burokratieprozessen besteht und das einzig erstrebenswerte die Pension danach ist.* *”*	*(W1. ID940)*
*“ich vielleicht auch zu wenig informtiert bin. Aber vor allem eintöniges Arbeiten und amtsärztliche (ergo nicht helfende?) Tätigkeit damit verbinde”*	*(W1. ID622)*
**Quotes stating why working in the PHS was considered attractive**	
**Good work-life balance and attractive working hours**	
*“familienfreundliche Arbeitszeit (modelle), keine Wochenend- oder Nachtdienste”*	*(W2. ID155)*
*“Familie wahrscheinlich besser mit dem Job vereinbar ist als im Schichtdienst im Krankenhaus”*	*(W2. ID156)*
**Addressing health challenges on a population level, on a systemic or structural level, or through prevention**	
*“ich gerne präventiv tätig wäre und nicht nur Schadensbegrenzung in den anderen Fächern betreibe”*	*(W2. ID106)*
*“Ich die Gesundheit von Menschen auf Bevölkerungsebene praeventiv und gesundheitsfördernd verbessern will”*	*(W2. ID118)*
*“der ÖGD aktuell nicht die Art Public Health fördert und ausfüllt, für die ich mich einsetzen will. Der ÖGD übernimmt viel mehr komplementäre Gesundheitsversorgung und bürokratische Aufgaben, als innovativ an der Verbesserung und Förderung der Bevölkerungsgeaundheit zu arbeiten und auf politischer Ebene dafür einzustehen”*	*(W1. ID973)*
**Having impact or making a difference**	
*“ich aktiv etwas für die Gesundheit größerer Personenkreise tun kann & nicht nur auf individueller Ebene”*	*(W2. ID130)*
*“man am Ende des Tages mit dem Gefühl etwas Gutes getan zu haben nach Hause gehen kann”*	*(W2. ID77)*
*“dieser für die Gesellschaft wichtige Aufgaben übernimmt”*	*(W1. ID327)*
*“ich Menschen* *helfen will* *”*	*(W1. ID693)*
*“Er müsste handlungsfähiger werden und mir glaubhaft machen, dass notwendige Entscheidungen auch wirklich effektiv getroffen und umgesetzt werden können”*	*(W1. ID718)*
**The occupation being exciting, diverse, or generally interesting**	
*“interssante abwechslungsreiche Arbeit mit großer Reichweite”*	*(W2. ID86)*
*“es ein großes Spektrum an Projekten gibt, in denen man sich engagieren kann und somit die Welt ein kleines bisschen verbessern kann”*	*(W1. ID291)*
*“ich die Arbeit so einschätzen würde, dass man sich nicht nur stumpf auf das eigene Fach beschränken muss, sondern auch verschiedene Aufgaben übernehmen kann”*	*(W2. ID121)*
**High degree of job security**	
*“Sicherheit bezüglich Arbeitsplatzgarantie und Vertragslaufzeit, Gehaltsstruktur, Arbeitszeiten, Relevanz der Tätigkeit”*	*(W2. ID34)*
**Quotes focusing on previous experience**	
*“Ich habe während der pandem. Sit. 2020–2021 im GA gearbeitet und war sehr enttäuscht von der eingefahrenen Struktur und der „Eingefahrenheit“ der Mitarbeiter*innen. Ich hoffe, dass mit einem Generationswechsel mehr Offenheit und Motivation im ÖGD Einzug hält. Es wurde leider nicht vorausschauend geplant. Motivierte und sehr gut qualifizierte Student*innen hatten keine Chance auf eine (langfristige) Anstellung oberhalb der Entgeltgruppe 4 (mit Masterabschluss) und wir haben uns alle andere Arbeitgebergesucht”*	*(W2. ID165)*
*“Ich hatte vor der Pandemie nur eine vage Vorstellung von der Arbeit der Gesundheitsämter. (Eben mit dem Klischee eines Jobs bei dem sich niemand überarbeitet). Aber dieses Vorurteil sehe ich nach einem Jahr Pandemie und Arbeit im Gesundheitsamt eben nicht nur bestätigt, sondern in fantastischem Ausmaß übertroffen. Ich bin ob des Gesamtauftretens des öffentlichen Gesundheitsdiensts während der Pandemie schockiert, enttäuscht und fassungslos”*	*(W2. ID114)*

All citations were left in their original form, including typos and emojis added by participants.
